# Functional PET/MRI reveals active inhibition of neuronal activity during optogenetic activation of the nigrostriatal pathway

**DOI:** 10.1126/sciadv.adn2776

**Published:** 2024-10-25

**Authors:** Sabrina Haas, Fernando Bravo, Tudor M. Ionescu, Irene Gonzalez-Menendez, Leticia Quintanilla-Martinez, Gina Dunkel, Laura Kuebler, Andreas Hahn, Rupert Lanzenberger, Bettina Weigelin, Gerald Reischl, Bernd J. Pichler, Kristina Herfert

**Affiliations:** ^1^ Department of Preclinical Imaging and Radiopharmacy, Werner Siemens Imaging Center, Eberhard Karls University Tuebingen, Tuebingen, Germany.; ^2^ Institute of Pathology and Neuropathology, Comprehensive Cancer Center, Eberhard Karls University of Tuebingen, Tuebingen, Germany.; ^3^ Cluster of Excellence iFIT (EXC 2180) “Image Guided and Functionally Instructed Tumor Therapies”, Eberhard Karls University of Tuebingen, Tuebingen, Germany.; ^4^ Department of Psychiatry and Psychotherapy, Medical University of Vienna, Vienna, Austria.; ^5^ Comprehensive Center for Clinical Neurosciences and Mental Health (C3NMH), Medical University of Vienna, Vienna, Austria.

## Abstract

The dopaminergic system is a central component of the brain’s neurobiological framework, governing motor control and reward responses and playing an essential role in various brain disorders. Within this complex network, the nigrostriatal pathway represents a critical circuit for dopamine neurotransmission from the substantia nigra to the striatum. However, stand-alone functional magnetic resonance imaging is unable to study the intricate interplay between brain activation and its molecular underpinnings. In our study, the use of a functional [fluorine-18]2-fluor-2-deoxy-d-glucose positron emission tomography approach, simultaneously with blood oxygen level–dependent functional magnetic resonance imaging, provided an important insight that demonstrates an active suppression of the nigrostriatal activity during optogenetic stimulation. This result increases our understanding of the molecular mechanisms of brain function and provides an important perspective on how dopamine influences hemodynamic responses in the brain.

## Teaser

Optogenetic functional PET sheds light on molecular mechanisms of fMRI neuronal activity.

## INTRODUCTION

The dopaminergic circuitry is instrumental in numerous essential functions within the nervous system, orchestrating processes related to motor control, reward processing, cognitive functions, and emotional regulation. Its dysfunction has been implicated in a variety of neurological and psychiatric disorders, including Parkinson’s disease (PD), schizophrenia, drug abuse, and attention-deficit hyperactivity syndrome (*1*). As one of the principal neuromodulatory systems in the brain, the dopaminergic system is subject of intense studies, and understanding this complex circuitry is pivotal for elucidating the underlying mechanism of these diseases and for developing targeted therapies. Consequently, the accurate characterization of this circuitry, including its biochemical, structural, and functional aspects, has become vital for both basic research and clinical applications, offering prospects for improved diagnostics and tailored interventions.

In vivo imaging technologies have greatly advanced our understanding of neuronal circuits at the whole-brain level. Two powerful noninvasive tools that have emerged in this field are positron emission tomography (PET) and functional magnetic resonance imaging (fMRI). Recent developments have led to the integration of PET and MRI into hybrid systems offering remarkable opportunities for comprehensive investigations (*2*–*7*). By integrating [^18^F]2-fluor-2-deoxy-d-glucose (FDG)–PET with blood oxygen level–dependent (BOLD)–fMRI, our approach leverages the strengths of both modalities to offer a perspective on the dynamic interactions within the dopaminergic system under optogenetic manipulation. This integration enables us to map out the metabolic and hemodynamic responses, providing a multifaceted view of the neuronal and physiological processes that underlie the functioning of the dopaminergic system, addressing some limitations of existing methods such as microdialysis, fast-scan cyclic voltammetry, fiber photometry, and [^11^C]raclopride PET. Microdialysis, while effective in measuring neurotransmitter levels in brain interstitial fluid, is hampered by low temporal resolution and spatial restriction to a single target area. Techniques such as fast-scan cyclic voltammetry and fiber photometry excel in real-time monitoring of dopamine release with high temporal resolution but are limited to small, localized regions. In contrast, our integrated [^18^F]FDG-PET/BOLD-fMRI approach provides noninvasive, whole-brain coverage, capturing dynamic changes of the dopaminergic system without disrupting tissue integrity. This method elucidates their impact on overall brain energy metabolism and blood flow, offering a holistic understanding of brain function. In addition, while [^11^C]raclopride, [^18^F]fluoro–l-dopa, and [^11^C]methylphenidate effectively assess dopamine receptor/transporter availability (*8*, *9*) and extracellular dopamine release (*10*), the tracers fall short in detailing downstream regional responses. By combining [^18^F]FDG-PET glucose metabolism measurements with BOLD-fMRI blood flow assessments, our approach elucidates the energetic and vascular underpinnings of dopaminergic neurotransmission, providing unprecedented detail and enhancing our understanding of the dopaminergic system. The insights gained from this research could advance our understanding of the molecular and systemic mechanisms of the dopaminergic system, with important implications for diagnosing and treating neuropsychiatric disorders.

There is a growing body of evidence suggesting a potential decoupling between metabolic and hemodynamic signals using simultaneously acquired PET/fMRI (*5*, *11*), implying that conventional imaging methods may fail to provide a comprehensive or completely accurate representation of the neuronal activity within the brain. This raises the fundamental question whether we can capture the complete picture of dopaminergic activity and its downstream pathways using stand-alone imaging techniques.

To explore this compelling question, we initiated a study using optogenetic stimulation (OGS), a cutting-edge method for precisely manipulating neuronal activity (*12*). We hypothesized that this approach might unveil hidden dimensions of neuronal functioning, including potential silencing mechanisms, which have been reported by other techniques (*13*), and that stand-alone imaging methods might overlook. BOLD-fMRI, with its superior spatial and temporal resolution compared to [^18^F]FDG-PET, which has been improved to study task-related brain activation in a single functional PET (fPET) session (*14*), each provides unique insights. Previous studies combining [^18^F]FDG-PET and BOLD-fMRI in rats during an electrical whisker stimulation paradigm revealed regional overlaps and mismatches in brain activation pattern between the two modalities (*15*, *16*). However, these data were not simultaneously acquired, as different stimulation paradigms and time points were used for PET and fMRI.

Here, we introduce an innovative approach, fusing OGS with fully simultaneous fPET/fMRI measurements in rats. Our findings not only enhance our understanding of inhibitory mechanisms during neuronal activation but also underscore the efficacy of hybrid PET/MRI systems in studying brain function. These insights offer an important contribution to the field, encouraging further exploration and refining our comprehension of the dopaminergic system.

## RESULTS

### BOLD signal suppression in dopamine-innervated brain regions during substantia nigra pars compacta stimulation

**Fig. 1.**
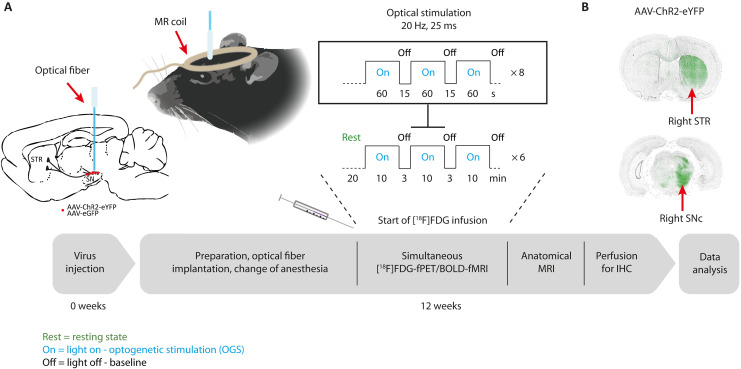
The time course and study protocol of simultaneous optogenetic [^18^F]FDG-fPET/BOLD-fMRI experiments are illustrated. (**A**) Adeno-associated virus (AAV)–ChR2 or AAV-GFP control virus was injected into the right SNc. Twelve weeks after viral vector injection, rats were catheterized and intubated. After fiber implantation, the [^18^F]FDG-fPET/BOLD-fMRI experiments were acquired using an [^18^F]FDG bolus-infusion protocol during optical stimulation of the SNc over 90 min using 6× 10-min stimulations and 3-min rest. Each 10-min stimulation block consisted of eight light-on and light-off phases. Within the on phase, a frequency of 20 Hz was set with a duty cycle of 50%, resulting in a pulse duration of 25 ms. After acquisition of an anatomical sequence, the rat brain was transcardially perfused for in vitro immunohistochemistry (IHC). (**B**) ChR2 and GFP control virus expression in the striatum (STR) and SNc was confirmed by fluorescence microscopy of ChR2–enhanced yellow fluorescent protein (eYFP) and enhanced GFP (eGFP).

To delineate the nigrostriatal system and investigate selective dopaminergic modulatory effects on neuronal activity and glucose metabolism, we performed simultaneous in vivo BOLD-fMRI/[^18^F]FDG-fPET scans in rats, overexpressing either channelrhodopsin-2 (ChR2) or green fluorescent protein (GFP) control virus in the right substantia nigra (SN) pars compacta (SNc), during OGS (Fig. 1, A and B). To allow for a within-group fPET data analysis, we applied [^18^F]FDG as a bolus plus constant infusion to maintain constant activity concentrations in the brain. This technique was first applied to identify task-relevant brain networks in human studies but has not been applied in rodent brain imaging yet (*16*, *17*). We started laser stimulation 20 min after beginning the simultaneous fPET/fMRI acquisition using a block design with 10-min stimulation blocks and 3-min rest between the blocks. We further divided each stimulation block into 60-s light-on and 15-s light-off periods.

fMRI data analysis was performed in ChR2- and GFP-injected animals by modeling each of the 10-min stimulation blocks using a canonical hemodynamic response function. For between-group analysis, a comparison between ChR2 and GFP animals was performed to control for nonspecific effects of the OGS. For the within-group analysis, the 10-min stimulation blocks were compared against 3-min baseline blocks.

**Fig. 2.**
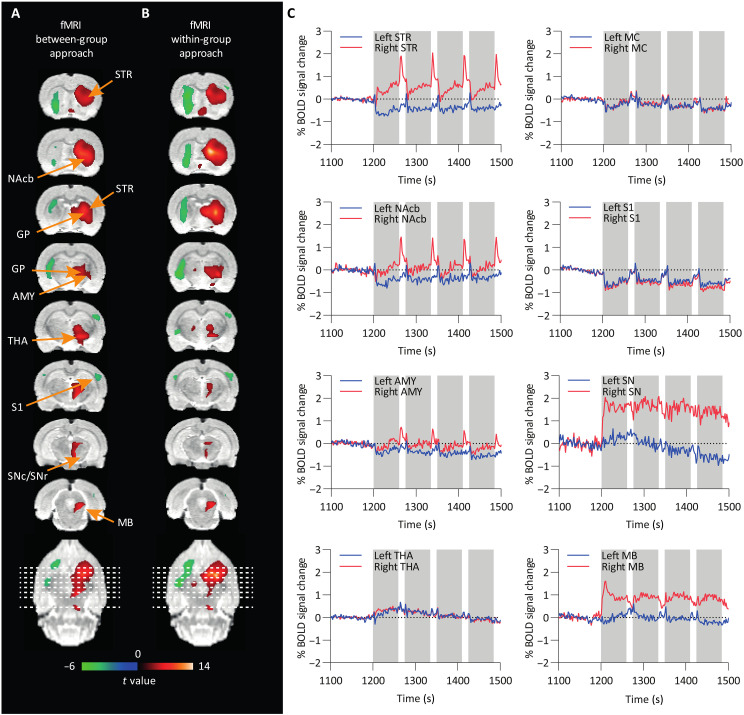
BOLD-fMRI *t*-activation maps reveal significant neuronal responses following OGS of the SNc. (**A**) Between-group [ChR2 (*n* = 18) versus GFP (*n* = 12)] and (**B**) within-group comparisons (ChR2, stimulation versus rest, *n* = 18) are shown. Positive (red) and negative (green) BOLD responses are shown (FWE-corrected *P* < 0.05 for cluster-level inference). (**C**) BOLD signal time courses from different brain regions. Gray bars indicate 60-s stimulation on periods. STR, striatum; AMY, amygdala; MB, midbrain; MC, motor cortex; S1, somatosensory cortex; NAcb, nucleus accumbens; THA, thalamus; GP, globus pallidus, SNc/SNr, substantia nigra pars compacta/pars reticulata.

OGS of nigrostriatal neurons resulted in positive BOLD-signal changes in brain regions of the basal ganglia, namely, the right striatum, nucleus accumbens, amygdala, thalamus, and midbrain, as well as negative responses in the left striatum and right and left somatosensory cortex (S1). Figure 2 (A and B) shows activated voxels presented as colored *t* maps overlaid on an MRI rat brain atlas after between-group (*n*_ChR2_ = 18 and *n*_GFP_ = 12) and within-group (*n*_ChR2_ = 18) analysis [at thresholds of *P* < 0.001 voxel level–uncorrected and *P* < 0.05 cluster-level family-wise error (FWE)–corrected]. A list reporting mean *t* values and the percentage of activated voxels within a region after cluster-level FWE correction at *P* < 0.05 is shown in Table 1 for between- and within-group analyses.

**Table 1.** Percentage of significant voxels per ROI and mean *t* values in fMRI. Results shown at uncorrected *P* < 0.001 with cluster-level FWE-corrected *P* < 0.05. Asterisk markings show areas with significant signal changes at voxel-level FWE-corrected *P* < 0.05. EC, entorhinal cortex; HIP, hippocampus; INS, insular cortex; OC, olfactory cortex; OFC, orbitofrontal cortex; PAG, periaqueductal gray; SC, superior colliculus; Sep, septum.

	Between-group approach positive BOLD		Between-group approach negative BOLD		Within-group approach positive BOLD in ChR2		Within-group approach negative BOLD in ChR2	
Brain region (ROI)	Activated voxels (%)	Mean *t*	Activated voxels (%)	Mean *t*	Activated voxels (%)	Mean *t*	Activated voxels (%)	Mean *t*
R AMY	2.0	3.9 ± 0.3			3.6	4.1 ± 0.4		
L AMY			0.2	3.4 ± 0.02			6.0	4.0 ± 0.2
R EC					0.01	3.7 ± 0.0		
L EC			0.1	3.6 ± 0.2			0.4	3.9 ± 0.2
L HIP anterior			0.9	3.6 ± 0.1			0.2	3.8 ± 0.1
R HIP posterior	0.4	4.2 ± 0.7						
R HYP	17*	4.9 ± 1.3			15*	4.5 ± 0.6		
R INS					0.1	3.9 ± 0.1		
L INS							0.3	4.1 ± 0.2
R MB	3.4*	4.7 ± 0.6			22*	5.3 ± 1.0		
L MC			0.1	3.5 ± 0.04			0.3	3.8 ± 0.1
R NAcb	2.2	3.7 ± 0.2			4.0	4.1 ± 0.3		
L NAcb			18	4.0 ± 0.3			18	4.4 ± 0.4
L OC			0.9	3.6 ± 0.2				
R OFC	0.2	3.6 ± 0.1			0.2	4.0 ± 0.2		
L OFC							1.1	4.1 ± 0.2
PAG	2.4	3.7 ± 0.2			2.7	4.1 ± 0.4		
R S1					0.02	4.1 ± 0.1		
L S1							1.1	3.9 ± 0.2
R SC					2.7*	4.9 ± 0.9		
Sep	17*	4.8 ± 1.0			33*	6.2 ± 2.1		
R SN	3.6*	5.0 ± 0.9			2.3	4.3 ± 0.4		
R STR	67*	5.0 ± 1.2			66*	5.3 ± 1.5		
L STR			31	3.9 ± 0.4			58	4.3 ± 0.4
R THA	53*	4.7 ± 0.7			40*	5.1 ± 1.2		
L THA					0.9	4.3 ± 0.5		

Mean % BOLD signal changes are shown over a period of 400 s for selected brain regions (Fig. 2C). Sixty-second stimulation blocks are highlighted in gray. We found positive BOLD signal changes in several areas of the basal ganglia including in the ipsilateral (right) striatum, nucleus accumbens, amygdala, and midbrain during the 60-s stimulation periods (on phase). However, the BOLD signal increase was rather low during stimulation, while we observed a robust BOLD signal overshoot after termination of the stimulation in the ipsilateral (right) striatum, nucleus accumbens, and amygdala, which went back to baseline within the 15-s rest period (off phase).

Negative BOLD signal changes appeared in the contralateral (left) striatum, nucleus accumbens, amygdala, ipsi- and contralateral motor cortex, and S1. Mean BOLD signal time courses of all ChR2 rats are shown over the whole scan time for selected regions in fig. S1. In GFP control rats, we did not observe responses to stimulation in the BOLD signal time courses (fig. S2A). The BOLD signal time course of one exemplary ChR2 and GFP rat is further plotted over the whole scan time in fig. S3A.

### Activation of the nigrostriatal pathway revealed by [^18^F]FDG-fPET within-group analysis

To directly compare hemodynamic responses from fMRI with metabolic responses from fPET, we established a within-animal PET analysis that allows us to compare activation versus baseline in the same animal. For this, we applied both the general linear model (GLM) described previously (*16*, *18*) and the independent component analysis (ICA) approach as within-sample methods to examine the OGS data. ICA has already been applied to [^18^F]FDG-fPET data to investigate brain glucose metabolism and connectivity during task-related designs (*17*, *19*). We used the aforementioned strategy (*19*) and further performed an automatic sorting of the resulting components based on spatial kurtosis (i.e., spatial sparseness), an approach that proved effective in isolating task-related components without the use of stimulus timing information (*20*–*22*). The OGS component map appeared as the first-ranked component with the highest kurtosis value (9.47), revealing significant [^18^F]FDG uptake during stimulation in the right SN, right midbrain, right thalamus, right hypothalamus, and right striatum. Additional parameters derived from component’s voxel value distribution, including skewness (a measure of the asymmetry of the distribution), spatial variability (a widespread/clustering measure), and frequency (the center of mass in spectral power), are shown in Table 2 (see table S1 for descriptive measures for all 20 components). Alternatively, the OGS component can also be identified for its lowest frequency content, following the power spectrum ranking method (*23*).

**Table 2.** ICA. Descriptive measures derived from the independent component’s voxel value distribution.

Component	Kurtosis	Skewness	Variability	Frequency
OGS (via highest kurtosis sorting)	9.4702	1.5296	0.89649	0.015126

**Fig. 3.**
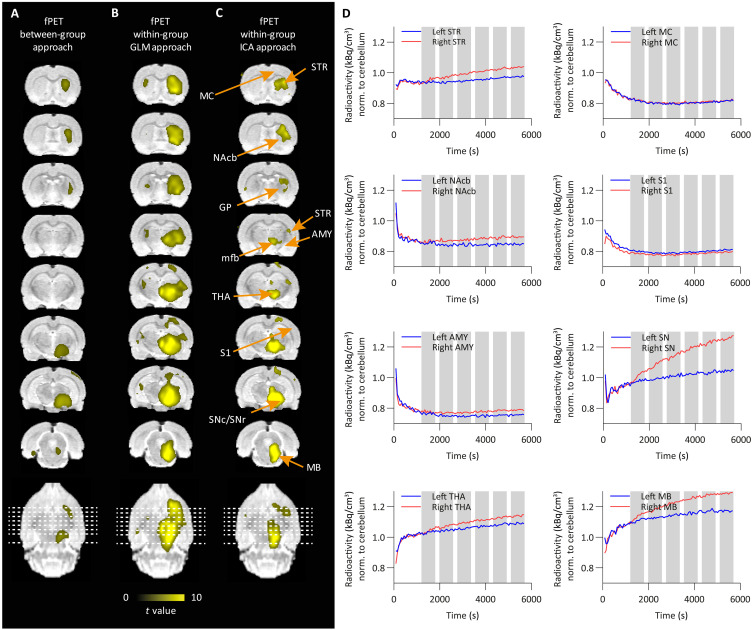
[^18^F]FDG-fPET *t* activation maps display metabolic responses following optogenetic SNc stimulation. (**A**) Between-group [ChR2 (*n* = 16) versus GFP (*n* = 14)], (**B**) GLM within-group (ChR2, stimulation versus rest, *n* = 16), and (**C**) ICA within-group (ChR2, stimulation versus rest, *n* = 16) comparisons are shown. Positive responses (yellow) are shown (FWE-corrected *P* < 0.05 for cluster-level inference). (**D**) [^18^F]FDG time-activity curves from different brain regions (gray bars indicate 10-min stimulation blocks). Right, ipsilateral; left, contralateral.

Figure 3 shows activated voxels as colored *t* maps overlaid on an MRI atlas using between-group (Fig. 3A), GLM within-group (Fig. 3B), and ICA within-group (Fig. 3C) analyses (*n*_ChR2_ = 16 and *n*_GFP_ = 14) of [^18^F]FDG-fPET data (at thresholds of *P* < 0.001 voxel level–uncorrected and *P* < 0.05 cluster-level FWE-corrected). In contrast to MRI, we did not observe any negative signal changes in the [^18^F]FDG-fPET data. A list reporting mean *t* values and the percentage of activated voxels within a region is shown in Table 3.

**Table 3.** Percentage of significant voxels per ROI and mean *t* values in fPET. Results shown at uncorrected *P* < 0.001 with cluster-level FWE-corrected *P* < 0.05. Asterisk markings show areas with significant signal changes at voxel-level FWE-corrected *P* < 0.05. AUD, auditory cortex; PG, pituitary gland; RS, retrosplenial cortex; V1, visual cortex.

	Between-group approach		Within-group GLM approach		Within-group ICA approach	
Brain region (ROI)	Activated voxels (%)	Mean *t*	Activated voxels (%)	Mean *t*	Activated voxels (%)	Mean *t*
R AMY	9.7*	4.6 ± 0.8	11*	5.0 ± 1.0	0.8	5.1 ± 0.9
R AUD			2.5	4.5 ± 0.6		
R EC	0.5	3.9 ± 0.4	1.0	4.4 ± 0.6		
R HIP anterior			5.0	4.2 ± 0.4	0.7	3.9 ± 0.2
L HIP anterior			6.1	4.1 ± 0.3		
R HIP posterior	13*	4.6 ± 0.8	34*	5.6 ± 1.2	6.0	4.8 ± 0.8
L HIP posterior			5.8	4.2 ± 0.4		
R HYP	24*	4.5 ± 0.7	48*	7.2 ± 2.3	36*	7.5 ± 3.0
L HYP			3.4*	5.4 ± 1.4	4.4	4.5 ± 0.5
R INS			10*	5.6 ± 1.8	2.7	4.8 ± 1.0
R MB	21.2*	4.4 ± 0.8	97*	7.7 ± 2.1	68*	7.1 ± 2.1
R MC			0.6	4.1 ± 0.3		
R NAcb			12	4.9 ± 0.8	2.3	4.3 ± 0.5
R OFC			7.1*	5.3 ± 1.2		
PG	3.9	3.9 ± 0.3	7.7*	5.2 ± 1.1	16	4.9 ± 1.0
R PAR			29	4.3 ± 0.4		
PAG	0.4	3.7 ± 0.2	26	5.0 ± 0.8	9.8*	5.1 ± 1.0
R RS			1.1	4.0 ± 0.3		
R S1			3.3	4.4 ± 0.5	0.5	4.4 ± 0.6
R SC			7.0	4.4 ± 0.6		
Sep			0.9	4.1 ± 0.3		
R SN	87*	5.8 ± 1.0	100*	10.0 ± 1.2	98*	9.8 ± 2.8
L SN			11	4.7 ± 0.7	1.9	4.8 ± 0.5
R STR	27*	4.3 ± 0.6	94*	6.5 ± 1.5	50*	4.9 ± 0.9
L STR			6.6	4.2 ± 0.3		
R THA	0.7	3.8 ± 0.3	74*	6.7 ± 2.0	24*	5.3 ± 1.3
L THA			4.1*/3.6	4.7 ± 0.8/3.9 ± 0.2	0.3	4.0 ± 0.1
R V1			2.7	3.9 ± 0.1		

During OGS, the GLM approach yielded increased [^18^F]FDG uptake in similar areas as after using the between-group and ICA within-group approaches (right SN, right midbrain, right hypothalamus, and right striatum). In addition, the GLM strategy showed strong uptake in the right insular cortex, thalamus, right hippocampus posterior, left hypothalamus, right orbitofrontal cortex, pituitary gland, and right amygdala.

Mean normalized time-activity curves of all ChR2-expressing rats are shown over the whole scan time for selected brain regions. Ten-minute stimulation blocks are highlighted in gray (Fig. 3D). We observed a gradual increase of [^18^F]FDG during the stimulation in the ipsilateral (right) striatum nucleus accumbens, thalamus, SN, and midbrain compared to the contralateral side, while little to no changes were observed in the amygdala, motor cortex, and S1. Mean normalized time-activity curves of all GFP-expressing rats are shown over 95 min for selected regions (fig. S2B). No changes between the left and right hemisphere of the selected brain regions were observed in the GFP group.

[^18^F]FDG activity of one exemplary ChR2 and GFP rat is plotted over the whole scan time in fig. S3B. [^18^F]FDG showed an increased accumulation in the ipsilateral (right) striatum compared to the contralateral (left) striatum in the ChR2 rat, while no differences between the ipsilateral (right) and contralateral (left) striatum were found in the GFP rat, confirming a stimulation-induced increase in [^18^F]FDG metabolism.

### Uncoupling of hemodynamic and metabolic responses to SNc stimulation

**Fig. 4.**
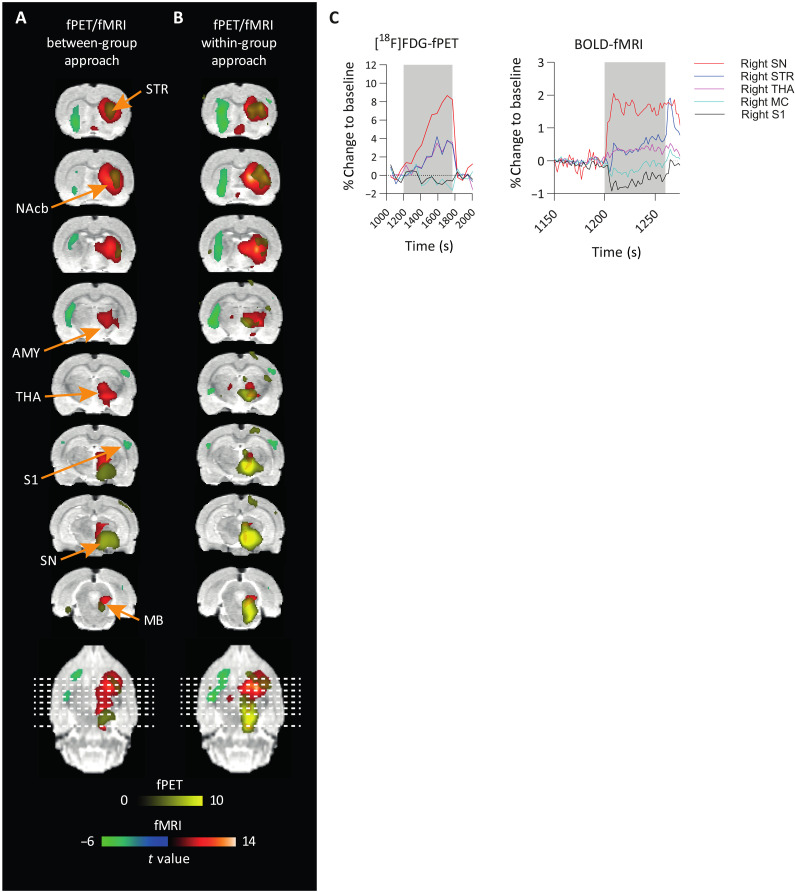
Overlay of [^18^F]FDG-fPET and BOLD-fMRI activation maps highlights the spatial and temporal differences in neuronal responses following OGS of SNc. (**A** and **B**) An overlay of significantly activated and deactivated areas (FWE-corrected *P* < 0.05 for cluster-level inference) after OGS of the SNc in fPET and fMRI is shown in colored overlays on a rat brain atlas. Activated areas in fMRI are depicted in red, deactivated areas in fMRI are depicted in green, and activated areas in fPET are depicted in yellow. The spatial extension of activated areas between modalities differs. (**C**) BOLD and [^18^F]FDG time courses in percent change from baseline, shown as mean values. A small stimulation block of 60-s on/15-s off for fMRI and a 10-min stimulation block for fPET are shown.

After validation of the within-group fPET analysis, we next aimed to compare hemodynamic and metabolic responses induced by nigrostriatal pathway activation. Figure 4 (A and B) shows a comparison of activated voxels from BOLD-fMRI and [^18^F]FDG-fPET as colored *t* maps overlaid on an MRI atlas (between- and within-group comparisons) (at threshold *P* < 0.001 voxel level–uncorrected and *P* < 0.05 cluster-level FWE-corrected). For within-group fPET, we show the results from the data-driven ICA approach (ICA with kurtosis/frequency sorting), which does not require a priori information, as is the case for the GLM fPET approach. Figure 4C shows a comparative analysis of the BOLD-fMRI and [^18^F]FDG-PET responses across five key regions during OGS of the SNc. This analysis offers insights into the distinct patterns of neural activation and suppression induced by dopaminergic modulation. PET and fMRI data revealed an immediate signal increase at the side of the stimulation in the SN, followed by delayed activations in the striatum and thalamus, contrasting with a negative BOLD-fMRI response in the primary motor cortex and S1.

Dice similarity coefficients were calculated to quantify overlapping voxels of both modalities (table S2). Within-group analysis revealed six overlapping regions: right striatum, right nucleus accumbens, right insular cortex, right thalamus, right S1, and right hypothalamus. Largest differences were observed in the spatial extension, most predominantly in the right striatum and right SN. Independent of the approach, BOLD-fMRI activation maps of the striatum show a larger spatial extension than fPET activation maps, while the opposite was observed in the SN. This is also confirmed when comparing the percentage of activated voxels per region presented in Tables 1 and 3 (within-group approach: striatum, 66% versus 50%; SN, 2.3% versus 98%). Coordinates of peak *t* values were extracted from regions of interest (ROIs) activated in both modalities to quantify the distance of the respective activation centers (table S2).

### Predictive neuronal activity in the SN revealed by multivariate pattern analysis

**Fig. 5.**
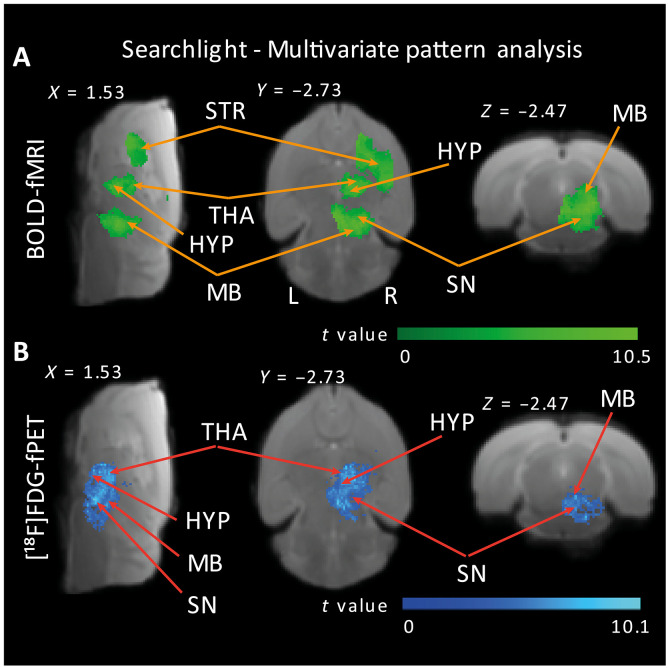
Classification searchlight analysis identifies brain regions where OGS is distinguishable from baseline activity across the whole brain. Colored voxels indicate centers of searchlights for the fMRI (**A**) and fPET (**B**) datasets, where the classifier could successfully discriminate between OGS and baseline signal patterns. Searchlight classification of >65% and whole-brain cluster-corrected *P* < 0.05 via comparison with 1000 random permutations. Sagittal, axial, and coronal views were selected to highlight the largest clusters containing information maps; for detailed results, see table S6. HYP, hypothalamus; L, left; R, right.

Following the minimal BOLD response in the SN yielded by the univariate within-sample analysis, we used multivariate pattern analysis (MVPA) to test whether sufficient information about the neural response to the OGS (versus baseline) would be contained in signal patterns of voxels within the SN. MVPA allows to evaluate differences between conditions with higher sensitivity than conventional univariate analysis by focusing on the analysis and comparison of distributed patterns of activity (*24*, *25*). We performed MVPA at a whole-brain level using SpaceNet classifiers (*26*) and searchlight (*27*) on both the fMRI and the fPET datasets. In addition, we conducted an ROI MVPA analysis that specifically targeted the SN. We tested the statistical significance of the prediction accuracy using a permutation test: Assuming that there is no class information in the data, the labels defining the conditions can be permuted without altering the expected accuracy using a given classifier and number of features (i.e., this would equal chance level). We performed 1000 permutations of a leave-one-subject-out cross-validated MVPA (*28*). The whole-brain fMRI and fPET MVPA results showed that information maps were distributed across right-lateralized voxels within the entire nigrostriatal pathway (table S5), with focal points including the SN, suggesting that the joint activity of voxels within the SN contained sufficient information about the OGS in both the fMRI and the fPET datasets (Fig. 5, fig. S7, and table S5). Further support was provided by the ROI-MVPA analysis centered on the SN, which yielded high prediction accuracies: 82.41% (*P* < 0.001) for the fMRI data and 69.27% (*P* < 0.001) for the fPET data.

Although to a lesser extent, discriminative information maps were also identified in left-lateralized regions such as the thalamus, hypothalamus, nucleus accumbens, and amygdala (table S6), confirming that the classifiers were not merely detecting univariate differences between the stimulation and baseline conditions. This indicates that the multivariate analyses exhibited greater sensitivity in discriminating between the stimulation and baseline categories.

To summarize, although the univariate fMRI analysis revealed a minimal BOLD response in the SN, multivariate pattern analyses substantiated that hemodynamic and glucose uptake signal changes within the right SN remained predictive and capable of distinguishing between OGS and baseline trials.

### Functional isolation of the striatum during OGS

Subsequent to the observation of a BOLD signal suppression during the OGS and a subsequent overshoot after stimulation cessation within the right striatum, nucleus accumbens, and amygdala (Fig. 2C), we carried out functional and molecular connectivity analyses to test whether the abovedescribed idiosyncratic signal changes could unveil a regulatory mechanism that would functionally isolate the striatum from other brain regions including areas upstream in the nigrostriatal pathway, in response to the OGS. We performed seed-based connectivity analyses (*29*) on both the fMRI and the fPET datasets to evaluate connectivity differences between OGS and baseline blocks.

**Fig. 6.**
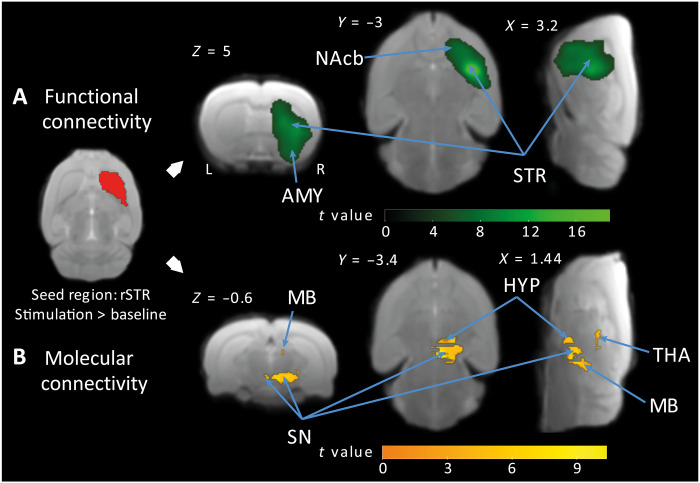
OGS of the SNc modulates functional and molecular connectivity within key brain regions. (**A**) Green color identifies voxels in the right striatum, nucleus accumbens, and amygdala, which exhibited stronger functional connectivity with the seed region (right striatum) for the contrast OGS versus baseline. (**B**) Yellow color identifies voxels upstream the nigrostriatal pathway showing a stronger metabolic coupling with the seed region for the contrast OGS versus baseline (results shown at FWE-corrected *P* < 0.05 for cluster-level inference).

Since the striatum receives axonal projections from SN dopaminergic neurons that corelease the two neurotransmitter γ-aminobutyric acid (GABA) and dopamine (*30*–*32*), possibly involved in the hypothesized isolation mechanism, we specifically sought to characterize connectivity differences in response to the OGS using the right striatum as the seed region. While the molecular connectivity analysis showed the expected strong metabolic coupling between the right striatum and upstream nigrostriatal pathway brain regions, such as the right thalamus, hypothalamus, midbrain, and SN during the OGS (compared to baseline), the functional connectivity analysis primarily revealed within-seed interactions (i.e., with voxels in the right striatum), extending to neighboring voxels in the nucleus accumbens and the amygdala. These results seem to evidence a metabolically active isolation mechanism, initiated by the OGS on areas receiving dopaminergic inputs, which could serve to functionally isolate the right striatum for areas upstream the nigrostriatal pathway (Fig. 6 and table S6).

### Reduced SNc activity confirmed by c-fos immunohistochemistry

**Fig. 7.**
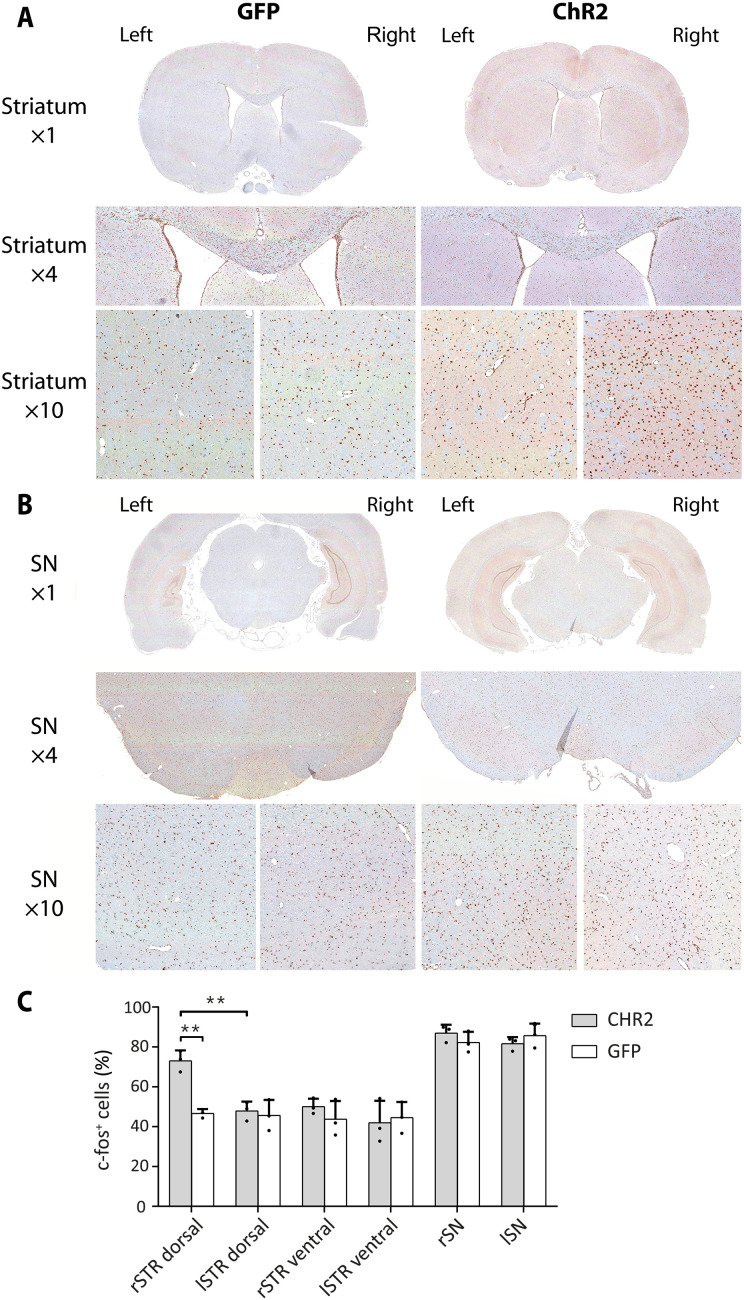
c-fos immunohistochemical staining reveals increased neuronal activation in the striatum but not SN following OGS. (**A**) c-fos staining in one exemplary ChR2 and GFP rat is illustrated for one selected region of the dorsal striatum in ×1, ×4, and ×10 magnifications. A higher number of c-fos^+^ cells were identified in the ×4 and ×10 magnifications of the right striatum of the exemplary ChR2 rat. (**B**) c-fos staining in one exemplary ChR2 and GFP rat is illustrated for one ROI of the SN in ×1, ×4, and ×10 magnifications. (**C**) The percentage of c-fos^+^ cells is increased in the right dorsal striatum of the three selected ChR2-expressing rats compared to the left dorsal striatum (*P* = 0.0036, Welch’s *t* test) of the ChR2-expressing rats and compared to the right dorsal striatum (*P* = 0.0061) of the three selected GFP-expressing rats. **P < 0.01.

To understand the mismatch between hemodynamic and metabolic responses in the two main regions, we performed c-fos^+^ stainings in the striatum and the SN. We found an increased c-fos expression in the right dorsal striatum of ChR2 rats compared to the left dorsal striatum and compared to ipsi- and contralateral dorsal striata of GFP rats (see Fig. 7A and fig. S4). However, no differences in c-fos expression levels were observed between the right and left SN (Fig. 7, B and C, and table S3). This result supports an inhibition of the neuronal activity in the SN during stimulation, a metabolically active process, resulting in high glucose demand.

### Exclusion of virus-induced neurotoxic effects via tyrosine hydroxylase staining

We further performed in vitro validation of neuronal integrity to exclude any viral- or stimulation-induced neurotoxicity in the SN and striatum. The tyrosine hydroxylase (TH) immunohistochemistry (IHC) in the SN revealed abundant TH^+^ neurons and its projections but without qualitative differences between the right and left sides in both GFP- and ChR2-expressing rats (fig. S5). In addition, massive presence of TH^+^ fibers was detected in the striatum, which is known to be a major postsynaptic target of the SN. In the striatum, the TH IHC did not reveal any qualitative differences in the dorsal/ventral and right/left striatum between GFP- and ChR2-expressing rats.

## DISCUSSION

fMRI and fPET are two valuable imaging techniques used in neuroscience research to study neuronal activation. In this study, we used an OGS of the dopaminergic pathway using simultaneous BOLD-fMRI and [^18^F]FDG-fPET imaging in the rat brain. The findings reveal important insights into both temporal and molecular aspects of brain function.

To enable within-group comparison of fPET data in rats, we used an [^18^F]FDG bolus + constant infusion protocol. In this study, we validated the application of ICA as a data-driven approach for analyzing rat fPET data acquired during OGS in rats. By automatically sorting components according to spatial kurtosis (*21*, *22*), we identified the [^18^F]FDG uptake map for the OGS component as the first kurtosis-ranked component. This was supported by a GLM-based approach, which identified consistent signal changes, thereby fostering efficient future studies by reducing the need for large control groups. Furthermore, we demonstrated the application of MVPA (*33*) on fMRI and fPET data, enabling the examination and comparison of distributed activity patterns to discern differences between conditions.

A distinct discovery of our study was that we observed a BOLD signal suppression during the stimulation and a subsequent overshoot after the cessation of the OGS within the right striatum, nucleus accumbens, and amygdala. Functional and molecular connectivity analyses provided evidence for a metabolically active isolation mechanism elicited in the aforementioned regions, which was modulated by the OGS. The fact that these brain areas are acknowledged recipients of dopaminergic inputs prompted us to suggest a potential role of dopamine in this mechanism.

Although MVPA showed that hemodynamic and glucose uptake signal changes within the right SN could discriminate between OGS and baseline trials, we observed only a minimal BOLD response in the SN in our univariate analysis when compared to fPET. To corroborate our observations, we conducted an ex vivo analysis of c-fos expression in both the striatum and the SNc. We found increased c-fos expression levels in the dorsal striatum, which receives input from the SNc, but we did not find discernible differences in the SNc. This occurred despite a high metabolic demand in the stimulated region. Dopamine release is modulated by numerous neuromodulators (*34*). One interpretation posits that OGS triggers the release of dopamine thereby activating dopamine D2 auto- and heteroreceptors and inhibiting further activation-induced dopamine release (*35*, *36*). This process may curtail the maximum attainable BOLD signal increase during stimulation (*13*) and may be important to regulate neurotransmitter levels at the synapse. This control is essential for the effective operation of the dopaminergic system. Upon termination of the stimulation, the presynaptic autoinhibition is lifted, leading to the BOLD signal overshoot. Although autoreceptors have been known for many years, the complexity of mammalian central nervous system circuits makes it difficult to isolate this mechanism from other neurotransmitter effects. In addition, several studies have shown that GABA is coreleased from dopaminergic neurons (*30*, *31*) and that this corelease acts as an early responder to dampen phasic-to-tonic dopamine signaling via ionotropic GABA type A receptors (*32*). Other models propose the role of GABAergic interneurons in autoinhibition by providing local feedback inhibition (*37*, *38*). Our observations of a relatively low BOLD response and an overshot immediately following the cessation of stimulation align with the autoreceptor model.

A key feature of the basal ganglia architecture is the division of incoming information into two distinct pathways: the direct and indirect pathways (*39*). Neurons in the direct pathway predominantly express the D1 dopamine receptor, whereas neurons in the indirect pathway express the D2 dopamine receptor (*40*, *41*). These receptors have opposing effects when bound to dopamine, influencing intracellular G protein messengers and subsequent changes in cellular excitation.

The classic theory, known as the “Go/No-go” model, posits that the direct and indirect pathways of the basal ganglia work antagonistically to facilitate and inhibit action, respectively (*42*–*44*). Conversely, other models suggest that the direct pathway selects the desired action, while the indirect pathway inhibits other competing actions, thereby emphasizing the chosen action (*45*–*47*). Each theory is supported by various behavioral, physiological, and imaging studies. Nevertheless, the exact mechanisms by which the direct and indirect pathways of the basal ganglia operate together still remain controversial and not fully understood (*48*).

Since dopamine has been shown to have a higher affinity to the dopamine D2 receptor (*K*_d_ = 25 nM) (*49*) than for the dopamine D1 receptor (*K*_d_ = 1.6 μM) (*49*), it is often assumed that they respond differently to tonic and phasic dopamine release (*41*, *50*). PET/MRI studies have shown that the combination of both makes up the fMRI signal (*51*, *52*). According to this model, low-affinity D1 receptors detect phasic, high-amplitude dopamine increases, while D2 receptors detect low-amplitude tonic dopamine increases (*53*). The negative BOLD response observed in the primary motor cortex and S1 in our study aligns with this model and may be explained by an enhanced inhibition of thalamic output due to a predominance of indirect pathway activation, which reduces cortical excitation. However, recent findings also show an activation of D2 receptors during phasic dopamine release (*54*, *55*). The impairment of dopamine and GABA corelease may also explain involuntary movements, so called l-dopa–induced dyskinesia, a side effect of long-term l-dopa treatment, where corelease of GABA is not achieved and D2 autoreceptors are not present on serotonergic neurons that take up the l-dopa due to the loss of dopaminergic neuronal projections (*56*, *57*). Thus, therapeutic strategies in PD should focus on restoring dopaminergic and GABAergic autoreceptor function in the striatum.

Alongside the positive BOLD signals, we observed stimulation-induced negative BOLD responses. These were not associated with a relative decrease in [^18^F]FDG in any brain region, a finding consistent with previous research in (*11*). While positive BOLD responses were accompanied by increased glucose metabolism, the authors observed that negative BOLD responses in regions of the default mode network did not show reduced glucose metabolism during a working memory task. Subsequent work demonstrated that this dissociation between negative BOLD response and glucose metabolism is dependent on the corresponding task-positive networks (*58*). Negative BOLD responses are considered to be a consequence of increased deoxyhemoglobin concentrations (*59*, *60*). These increases in deoxyhemoglobin occur during increased oxygen consumption compared to a constant cerebral blood flow or during decreased cerebral blood flow compared to a higher, stable, or only slightly reduced oxygen consumption. The precise physiological origin of the negative responses remains debated with theories including the “vascular steel” effect (*61*, *62*), the “vascular sharing” effect (*63*–*65*), and regional extremely high oxygen consumption resulting from strong neuronal activation that cannot be balanced by cerebral blood flow increases (*62*). In addition, neurotransmitter release might provoke neurovascular responses that can eventually affect the BOLD signal (*66*, *67*). A recent study further suggests that opioidergic neurotransmission contributes to negative BOLD-fMRI signals in the striatum (*68*). We hypothesize that neurotransmitter and vasoactive effects play a crucial role in the positive and negative responses, but further studies are needed to pinpoint the exact molecular mechanisms.

We further observed metabolic and hemodynamic changes in similar regions, yet differences were found in the spatial extent, location of the regional activation center, proportion of overlapping voxels, and significance of activated regions between the two modalities. For instance in the within-group approach, BOLD-fMRI revealed 15 activated brain regions and 9 regions with a negative BOLD response, while [^18^F]FDG-fPET revealed 16 activated brain regions. Of these regions, nine showed activation in both modalities, with six regions having overlapping voxels. The observed discrepancies between the two modalities can be attributed to their distinct physiological readouts. While [^18^F]FDG is a marker of glucose consumption (metabolic response), the BOLD signal is driven by localized changes in blood flow and blood oxygenation (hemodynamic response). Although several studies indicate the highest glucose consumption in neurons at the synaptic level, our data also support high glucose consumption at the soma. In contrast to PET, MRI enables in vivo imaging with higher spatial resolution, typically in the range of 0.27 mm by 0.27 mm in plane for the applied echo planar imaging–BOLD sequence. However, vascular effects are not confined to the activation site, and larger vessels contribute more to the BOLD signal. Consequently, there is a widespread effect that limits spatial resolution and may result in a mislocalization of activation centers (*69*).

Our findings, while primarily focused on understanding the broader impact of nigral activity on brain function, also have potential implications for the diagnosis and treatment of PD. The ability of [^18^F]FDG-PET to capture metabolic changes across the entire brain, beyond the striatum, provides valuable insights into the systemic effects of nigral-striatal dysfunction, which are characteristic of PD. While traditional dopaminergic tracers such as fluoro–l-dopa and dihydrotetrabenazine are highly effective in visualizing dopaminergic pathways, they predominantly highlight striatal changes and may overlook the broader network disruptions that contribute to the full spectrum of PD symptoms. Our results suggest that [^18^F]FDG-fPET, by providing a more comprehensive view of brain metabolism, could complement existing diagnostic tools by identifying changes in regions such as the thalamus, amygdala, and cortex, which are also involved in the disease process. Furthermore, understanding the metabolic consequences of dopamine and potentially GABA release in these regions could inform the development of new therapeutic strategies aimed at modulating activity across these broader networks, rather than focusing solely on the nigrostriatal pathway. This could lead to more effective treatments that address both motor and nonmotor symptoms of PD, ultimately improving patient outcomes.

One notable limitation of our study relates to the utilization of anesthesia, a common element in preclinical imaging studies. Anesthesia has the potential to affect vascular and metabolic responses, which can subsequently lead to alterations in the responsiveness to neuronal stimulations. Although medetomidine anesthesia has been proposed for small animal fMRI experiments in earlier research (*70*–*72*), it may elevate blood glucose levels (*73*, *74*), reducing the uptake of [^18^F]FDG into the brain (*74*, *75*). Isoflurane, often used in [^18^F]FDG-PET experiments, can also substantially influence the BOLD signal owing to its vasodilatory effect (*76*–*79*). Thus, in an effort to optimize the methodological approach, we selected α-chloralose anesthesia. This choice is based on its suitability for both modalities and its strong functional-metabolic coupling effects, which induce robust fMRI-BOLD activations even after weak stimulations, making it appropriate for [^18^F]FDG-fPET imaging (*80*–*82*). However, older studies in *Xenopus* oocytes suggest that α-chloralose may potentiate GABA-induced currents, presumable through an interaction with GABA type A receptors (*83*, *84*). Since GABA has been shown to be coreleased from dopaminergic nerve terminals, it may induce an enhanced modulation on GABAergic activity that may be less pronounced in a conscious state. In addition, an older study in rats did not find an influence of α-chloralose on dopamine content and metabolism in the rat striatum (*85*). In the context of our study, we strived to mitigate potential confounding effects through consistent anesthesia protocols and by contrasting our stimulations to resting state periods in the same animals. Future studies designed to explore these interactions would be very valuable.

The present study identified pronounced activations in regions within the basal ganglia circuitry, including the striatum, thalamus, and cortex. We also detected increased activation in areas such as the amygdala, septum, hippocampus, periaqueductal gray, and orbitofrontal cortex. These findings might be attributed to the partial stimulation of ventral tegmental area dopamine neurons (*1*). In addition, our study did not involve the use of TH- or dopamine transporter (DAT)–Cre rats for selective dopaminergic stimulation. This means that OGS may have incidentally extended to areas such as the SN pars reticulata, located beneath the SNc.

Another limitations of our study is the use of a relatively long and intense stimulation protocol, which was chosen on the basis of pilot experiments to optimize signal detection in our [^18^F]FDG-PET imaging. While this approach allowed us to capture robust metabolic changes across multiple brain regions, the strong and sustained nature of the stimulation may not accurately reflect physiological conditions encountered in typical brain function. This could potentially lead to exaggerated responses that might not be representative of normal dopaminergic activity. In addition, the prolonged stimulation differs from the shorter protocols commonly used in fMRI studies, which may limit the direct comparability of our findings with those obtained from other imaging modalities. These factors should be considered when interpreting the generalizability of our results, and future studies may benefit from exploring a range of stimulation intensities and durations to better understand their effects on brain function and metabolism. Furthermore, our study did not directly address how these findings apply to the clinical diagnosis and treatment of PD, which may require additional research using disease-specific models and parameters.

In conclusion, our study sheds light on the intricacies of the dopaminergic pathway, providing insights into the relationship between BOLD signals and metabolic responses. By using simultaneous optogenetic BOLD-fMRI and [^18^F]FDG-fPET imaging, we were able to observe a complex interaction involving both hemodynamic and metabolic processes. This integrated approach may help in identifying specific patterns of brain activation. One such example could include the effects of deep brain stimulations in patients with PD, in which targeting the classical regions has been shown to be insufficient for improving the entire spectrum of PD symptoms in addition to the observed side effects (*86*). Using simultaneous [^18^F]FDG-PET/BOLD-fMRI during neuronal stimulations may help to identify target regions to improve motor and nonmotor symptoms in PD. The findings and the application of cutting-edge techniques in this research offer a roadmap for future investigations into brain function. While the present study unveils the potential of these methods and uncovers interesting aspects of the neuronal mechanisms, it also emphasizes the need for further detailed research to unravel the complexities of the mammalian central nervous system circuits. Our results not only enhance the current understanding of the brain’s neurotransmitter systems but also pave the way for more focused and nuanced explorations, especially regarding the interactions between different neurotransmitters and their effect on overall brain functionality.

## MATERIALS AND METHODS

### Experimental timeline

Figure 1A shows a simplified time course of the experimental procedures. We randomly divided the rats into two groups and injected an adeno-associated virus (AAV) vector containing either ChR2 (*n* = 21) or AAV-GFP (*n* = 15) rats into the right SNc. Twelve weeks after viral vector injection, we implemented an optical fiber above the SNc and performed simultaneous [^18^F]FDG-fPET/BOLD-fMRI scans. We started the laser stimulation 20 min after start of the fPET/fMRI acquisition using a block design with 3-min rest between the blocks. The stimulation protocol used in this study was based on a study by Bass and colleagues (*87*) in which maximal peaks for dopamine release were observed at 20-, 30-, and 40-Hz frequency with 20-, 10-, and 4-ms light pulse widths, respectively. To achieve robust, reliable, and reproducible [^18^F]FDG-PET and BOLD-fMRI signal response, we used long stimulation durations and high power.

We divided each 10-min stimulation block into 60-s on and 15-s off stimulation phases. Light frequency within the on phases was 20 Hz with a duty cycle of 50% and a resulting pulse duration of 25 ms. After the acquisition, we performed an anatomical MRI, subsequently transcardially perfused the rats, and harvested the brains for in vitro validation. We performed fluorescence microscopy to confirm ChR2–enhanced yellow fluorescent protein (eYFP) and enhanced GFP (eGFP) viral vector expression in the striatum and SNc (Fig. 1B).

### Animals

We conducted all animal experiments in compliance with the European directives on the protection and use of laboratory animals (Council Directive 2010/63/UE), with the German animal protection law, and with approval of the official local authorities (Regierungspräsidium Tübingen, permit number R 6/17). Male Long-Evans (*n* = 36) rats were purchased from Charles River Laboratories (Calco, Lecco, Italy). All rats were maintained in our vivarium on a 12:12-hour light-dark cycle and were kept at a room temperature with 40 to 60% humidity. Rats had free access to a standard diet and tap water.

During the time course of the experiment, nine rats were excluded from the data analysis due to technical or experimental failures: fiber implantation (*n* = 3), PET insert (*n* = 3), and MR (*n* = 3). During the study, the frequency bandwidth was changed because of a gradient coil exchange: 3 GFP and 5 ChR2 rats were scanned with a frequency bandwidth of 166,666.7 Hz; 9 GFP and 13 ChR2 rats were scanned with a frequency bandwidth of 119,047.6 Hz.

### Stereotaxic viral vector injection

Before the viral vector injections, we allowed the rats to adapt for at least 2 weeks in the animal facility. We anesthetized each rat (*n* = 36,375 ± 27 g) with an intraperitoneal injection of a mixture (1 ml/kg) of fentanyl (0.005 mg/kg), midazolam (2 mg/kg), and medetomidine (0.15 mg/kg). The head was shaved, and the animal was placed into a stereotaxic frame. A central incision was made to expose bregma and lambda. A 5-ml Hamilton syringe needle (Hamilton Company, Reno, NV, USA) was enclosed by a glass capillary (inner diameter, 50 ± 5 μm; Hilgenberg GmbH, Malsfeld, Germany). Stock solutions of pAAV-hSyn-hChR2(H134R)-EYFP (#26973; AAV5, 1.7 × 10^13^ gene copies/ml) or pAAV-hSyn-EGFP (#50465; AAV5, 1.2 × 10^13^ gene copies/ml) (Addgene Inc., Watertown, MA, USA) were diluted to 8.5 × 10^11^ gene copies/ml using phosphate-buffered saline (PBS) (Gibco, Life Technologies Inc., Carlsbad, CA, USA). Two microliters were slowly injected (0.1 μl every 15 s) through a drill hole into the right SNc [mediolateral, −2.0 mm; anterior-posterior, −5.0 mm; dorsoventral, −7.2 mm; according to the stereotaxic atlas of Paxinos and Watson (*88*)]. To allow for diffusion of the virus into the tissue, the needle was left in place for 5 min. Before slowly retracting the needle from the brain (3.5 mm/min), it was withdrawn to −7.0 mm (dorsoventral) for another 2 min. The incision was closed by four to five stitches, and a subcutaneous antidote injection of atipamezol (0.75 mg/kg) and flumazenil (0.2 mg/kg) was administered.

### Simultaneous [^18^F]FDG-fPET/BOLD-fMRI with OGS

#### Optical setup

A 473-nm laser (MBL-III–473 nm–100 mW, PhotonTec Berlin GmbH, Berlin, Germany) with a maximum output power of 100 mW equipped with an FC/PC fiber coupler having a numerical aperture of 0.22 was used for OGSs. The laser was connected (FC/PC MM Fiber Connector, 230 μm, Stainless Steel, Thorlabs, Newton, NJ, USA) to an approximately 6-m-long optical fiber (TECS-Clad multimode optical fiber, Thorlabs, Newton, NJ, USA) with a glass fiber core of 200 μm and a numerical aperture of 0.39. The FC/PC connector was assembled and polished in-house using four different polishing sheets sequentially: silicon carbide lapping 5-μm grit, aluminum oxide lapping 3- and 1-μm grit, and calcinated alumina lapping 0.3-μm grit (Thorlabs, Newton, NJ, USA). The implantable end of the fiber was stripped for at least 2.5 cm, and a ceramic ferrule (2.5-mm multimode ceramic ferrule and 231-μm bore size, Thorlabs, Newton, NJ, USA) was glued (LOCTITE 454, Henkel AG & Co. KGaA, Dusseldorf, Germany) around the bare fiber end. After drying, the length of the protruding fiber was cleaved to a length of at least 8.2 mm. The laser was coupled to a power supply unit (PSU-III-LED, PhotonTec Berlin GmbH, Berlin, Germany) with transistor-transistor logic (TTL) modulation up to 1 kHz. It was driven by a stimulus generator (STG 2004, Multi Channel Systems MCS GmbH, Reutlingen, Germany) controlled by a flexible software (MC_Stimulus II, Multi Channel Systems MCS GmbH, Reutlingen, Germany). Fiber output power was measured using a fiber optic power meter (PM20A, Thorlabs, Newton, NJ, USA) before each single scan.

### Animal preparation

AAV-injected rats (*n*_ChR2_ = 21, *n*_Ctrl_ = 15, 12 ± 1 weeks after surgery, 510 ± 36 g) were fasted overnight. Anesthesia was induced with 5% isoflurane evaporated in air in an induction chamber. After loss of the righting reflex, isoflurane was maintained at 2.5 to 3% evaporated in air at a flow rate of 0.8 liter/min. The head was shaved, and a blood sample was collected by puncturing the tail vein for glucose determination (124 ± 13 mg/dl). One tail vein catheter was placed on each side for anesthesia and tracer infusions. Endotracheal intubation was performed using a self-made cannula and an external light source for correct placement of the tube. The small animal ventilator (DC1 73-3629, Harvard Apparatus, Holliston, MA, USA) was set to 60 breaths/min with an inspiration duration of 60% of the ventilation cycle. The end inspiratory pressure was set to approximately 12-cm H_2_O and the flow to 500 ml/min. During preparation and surgery, animals were warmed by a heating pad.

### Optical fiber implantation

The rat was placed into a stereotaxic frame. A central incision was made to expose bregma and lambda. The optical fiber was inserted through a drilled hole into the right SNc (mediolateral, −2.0 mm; anterior-posterior, −5.0 mm; dorsoventral, −7.1 mm; according to the stereotaxic atlas of Paxinos and Watson). Superglue was applied to fixate the ceramic ferrule to the skull. Isoflurane levels were slowly reduced after an initial bolus of 16 mg of α-chloralose (Sigma-Aldrich Chemie GmbH, Taufkirchen, Germany), followed by another bolus containing 5 mg of α-chloralose and 0.25 mg of pancuronium bromide (Inresa Arzneimittel GmbH, Freiburg, Germany). A constant infusion of α-chloralose (20 mg/kg per hour) and pancuronium bromide (1 mg/kg per hour) was started and maintained during the whole time course of the experiment along with 0.5% isoflurane evaporated in air.

### [^18^F]FDG-fPET/BOLD-fMRI

[^18^F]FDG was synthetized using [^18^O]water and the ^18^O(p,n)^18^F nuclear reaction described elsewhere (*89*). Simultaneous fPET/fMRI experiments were performed on a small animal 7-T MRI system (ClinScan, Bruker BioSpin MRI GmbH, Ettlingen, Germany) equipped with a small-animal PET insert, as previously described (*90*). A linearly polarized radio frequency coil (Bruker BioSpin MRI GmbH, Ettlingen, Germany) with an inner diameter of 72 mm was used for signal excitation and a planar single loop surface coil with an inner diameter of 20 mm (Bruker BioSpin MRI GmbH, Ettlingen, Germany) was used as receiver coil. Rats were placed on a water-heated bed (MedRes, Cologne, Germany), connected to the small animal ventilator (DC1 73-3629, Harvard Apparatus, Holliston, MA, USA) and to a feedback temperature control unit (MedRes, Cologne, Germany) set to 36.5°C. The temperature was constantly monitored by a rectal probe; oxygen saturation and heartbeat were monitored using a MR compatible pulse oximeter (Bruker BioSpin MRI GmbH, Ettlingen, Germany).

Localizer images were acquired to position the rat brain in the PET/MRI center of the field of view. B0 shimming was performed to optimize magnetic field homogeneity. After an isoflurane washout period of at least 1 hour, the PET insert and a T2*-weighted gradient echo planar imaging sequence (duration, 5700 s; echo time, 18 ms; repetition time, 2000 ms; voxel size, 0.27 mm by 0.27 mm by 1.00 mm; field of view, 25 mm by 19 mm; image dimensions, 92 pixels by 70 pixels by 20 pixels; slice thickness, 0.8 mm; slices, 20) covering the brain were started simultaneously. A total of 141 ± 8 MBq of [^18^F]FDG were injected 30 s after the start of the fPET and fMRI acquisition using a bolus (167 μl/min for 1 min) plus constant infusion (6.7 μl/min for 93.5 min) protocol. Dynamic PET data were acquired for 95 min and saved as list-mode files. Laser stimulation was started 20 min after start of the simultaneous fPET/fMRI acquisition using a block design described above. Laser irradiance values of 20 ± 3 mW were measured in continuous mode before each fiber implantation using the fiber optic power meter.

At the end of the scan, an anatomical T2 TurboRARE sequence was acquired (echo time, 67 ms; repetition time, 1800 ms; rare factor, 28; averages, 1; field of view, 40 mm by 32 mm by 32 mm; image dimensions, 160 pixels by 128 pixels by 128 pixels; voxel size, 0.25 mm by 0.25 mm by 0.25 mm). To allow for maximal c-fos expression, the animal was transcardially perfused with 50 ml of PBS at room temperature, 50 ml of PBS cooled to 4°C, and 50 ml of 4.5% paraformaldehyde (SAV Liquid Production GmbH, Flintsbach am Inn, Germany) 90 min after the start of the first stimulation phase. A second blood sample was collected from an intrathoracic vein for glucose determination (86 ± 10 mg/dl) right before perfusion. The brain was surgically removed and fixed in 4.5% formalin (SAV Liquid Production GmbH, Flintsbach am Inn, Germany).

### Imaging data analysis

#### Data preprocessing

fPET list-mode data were divided into 95× 1-min time frames. Sinograms were reconstructed into a dynamic fPET image using OSEM2D reconstruction algorithm. The dynamic brain fPET scans were converted into Neuroimaging Informatics Technology Initiative (NIfTI) format using PMOD software. fMRI and anatomical images were converted into NIfTI format using Bruker2NIfTI software (v1.0.20170707, Sebastiano Ferraris, University College London).

Data preprocessing was conducted as previously described (*5*) using Statistical Parametric Mapping 12 (SPM 12; Wellcome Trust Centre for Neuroimaging, University College London, London, UK) via MATLAB (The MathWorks, Natick, MA, USA) and Analysis of Functional NeuroImages (National Institute of Mental Health, Bethesda, Maryland, USA). In summary, realignment of fMRI and fPET data was performed in SPM. Binary masks were generated from average images and the anatomical MRI scans. With these, the brain was extracted from the fPET, anatomical reference, and fMRI image (“skull stripping”) before coregistration of the fPET and fMRI to the anatomy. Spatial normalization was performed using parameters, which were calculated by comparing the anatomical reference to the Schiffer rat brain atlas (*91*). The normalized fMRI and fPET images were smoothed using a 1.5-mm by 1.5-mm by 1.5-mm Gaussian kernel toward the spatial resolution of the PET insert. A temporal high-pass filter with a cutoff frequency of 256 Hz was applied to the fMRI data, with the purpose of removing scanner-attributable low-frequency drifts in the fMRI time series. Although SPM’s default high-pass cutoff is set to 128 Hz, we increased the cutoff frequency to 256 Hz, since this strategy has been proposed to improve the signal-to-noise ratio when using block lengths of more than 15-s off duration as is the case in the present study (*92*).

Extraction of mean time courses within an ROI was performed using MarsBar (*93*). The list of 54 selected ROIs, including abbreviations and volumes, is included in table S4.

#### fMRI statistical analysis

Data were analyzed using SPM version 12 (www.fil.ion.ucl.ac.uk/spm). A block design was used for the ChR2 and GFP groups (*92*), modeling each of the six 10-min stimulation blocks using a canonical hemodynamic response function that emulates the early peak at 5 s and the subsequent undershoot (*94*). The within-subject design matrix for the first level analysis included two regressors: OGS and baseline (3 min between stimulation blocks). Two contrast images per individual were calculated as follows: OGS > baseline and baseline > OGS.

##### Between-group approach

Single mean images for each contrast of interest (OGS > baseline and baseline > OGS) were first generated for each subject. Then, a two-sample *t* test was carried out to identify the regions that showed significant signal changes between the ChR2 and the GFP groups. Results were thresholded at *P* < 0.001 for voxel-level inference with a cluster-level threshold of *P* < 0.05 corrected for the whole-brain volume using FWE, which controls for the expected proportion of false-positive clusters. This threshold was also chosen for all subsequent fMRI and fPET analyses to ensure consistency and simplify the interpretation. Furthermore, this approach has been shown to provide control of the false-positive rate comparable to other software programs (*95*). Nevertheless, results also indicate significant findings after correction at *P* < 0.05 FWE voxel level, which is highly conservative.

##### Within-group approach

Single-subject voxel-wise statistical parametric maps for the aforementioned contrasts were obtained and subjected to group-level one-sample *t* tests. The significant map for the group random effects analysis was thresholded at *P* < 0.001 for voxel-level inference with a cluster-level threshold of *P* < 0.05 (FWE-corrected).

#### fPET statistical analysis

##### Between-group approach

[^18^F]FDG-fPET images were first subjected to intensity normalization with reference to the cerebellum (*96*). A two-sample *t* test was used to compare changes in glucose metabolism induced by OGS during the last 10-min stimulation block (corresponding to fPET frames 86 to 95 = time window with onset at second 2551 with a duration of 600 s) between GFP and ChR2 rats. Results were thresholded at *P* < 0.001 for voxel-level inference with a cluster-level threshold of *P* < 0.05 (FWE-corrected).

##### Within-group GLM approach

Modeling of [^18^F]FDG-fPET data with the GLM was done in MATLAB as described previously (*16*, *18*). Before the GLM, the signal-to-noise ratio of fPET data was increased by application of a low-pass filter with a cutoff frequency of ^1^/_5_ min (i.e., half the duration of the task block). The GLM is then used to separate task effects from baseline by construction of a design matrix that models task effects. This approach is most similar to conventional fMRI analyses (see within-group fMRI statistics above), thus yielding the term fPET. The design matrix included an OGS regressor and one for the baseline. We defined the OGS regressor as a ramp function with a slope of 1 kBq per frame when stimulation was active and zero otherwise. This is equivalent to the integral of a boxcar function used in fMRI task analyses. The baseline regressor accounts for the continuous uptake of the radioligand due to its irreversible kinetics. We defined it as the average of all gray matter voxels, excluding those voxels declared as activated with the fMRI within-group approach. This approach has been shown to be the best choice in terms of model fits (*18*), yielding comparable results to an independent baseline definition (*4*), and does not affect test-retest reliability (*97*). That is, each voxel’s time course was modeled by two regressors, namely, the OGS time course and the baseline signal, thus isolating stimulation effects from the raw fPET time-activity curve. The resulting β values of the OGS regressor were then subject to group-level statistical analysis in SPM at *P* < 0.001 for voxel-level inference with a cluster-level threshold of *P* < 0.05 (FWE-corrected).

##### Within-group ICA approach

The data-driven ICA approach is a method for recovering underlying signals from linear mixtures of those signals, which draws upon higher-order signal statistics to estimate a set of components that are maximally independent from each other (*98*). ICA separates sources by maximizing their non-Gaussianity, and, therefore, non-Gaussianity is fundamental for ICA model estimation (*99*). One way to understand the connection between independence and non-Gaussianity is offered by the central limit theorem, which states that the distribution of a sum (or mixture) of random variables tends to be more Gaussian than the original random variables. This, in turn, implies that when the sources are made more non-Gaussian, they become more independent (or unmixed). The distance to a Gaussian can be approximated using measures of non-Gaussianity, such as skewness and kurtosis, the latter being widely used for estimating non-Gaussianity in ICA. ICA algorithms, including FastICA and Infomax, maximize independence by finding components that have either maximum or minimum kurtosis (*100*, *101*).

In the case of fPET, ICA first requires a preprocessing step to remove the global baseline signal before the unmixing stage. This technique is conducted to improve the sensitivity for an accurate inference of spatially independent components. Following the procedure described in (*17*, *19*), we first applied whole-brain normalization to obtain four-dimensional volumes that represented the dynamic relative [^18^F]FDG uptake (time-activity) map (*96*). Two further preprocessing steps were implemented before the application of ICA: data reduction and whitening. Data reduction was performed by principal components analysis to capture most of the variability in the data (>99%) while reducing its dimensionality. Prewhitening was done to improve the convergence of the ICA algorithm and was achieved simultaneously with principal components analysis. To separate the independent components, we used the FastICA algorithm (*100*, *102*, *103*). We estimated 20 components per subject, as this number provided a reasonable trade-off between preserving most of the variance while considerably reducing the size of the data. Group-level spatial ICA was conducted using temporal concatenation, which is a widely used approach in group fMRI (*102*) and which has already been successfully applied to fPET data (*17*, *19*). The resulting components were sorted according to spatial kurtosis (i.e., a measure of the sparseness of a distribution) following the general framework presented by Lu and Rajapakse (*22*). ICA was implemented with the GIFT v4.0b (*102*) and CONN v22a (*104*) toolboxes in MATLAB v.R2019a (Natick, MA, USA).

While the GLM approach uses a model-based hypothesis, ICA is data-driven and does not require a priori assumptions on the form and shape of the expected [^18^F]FDG-fPET response. On the other hand, the GLM is simpler to implement and interpret, whereas the ICA approach requires a posteriori selection of components, which can be challenging when the spatial distribution of the effects is unknown. Here, we overcome the need for a manual identification of task effects by automatically sorting components according to spatial kurtosis (*21*, *22*).

#### Percent-overlap-of-activation 

To evaluate the percent overlap of activation between the fPET and fMRI results, we used the reliability measure proposed by Rombouts *et al.* (*105*) and Machielsen *et al.* (*106*), which is identical to the similarity coefficient proposed by Dice (*107*). According to this measure, the overlap of activation for any two replications (e.g., *k* and *m*) is established as in Eq. 1, where *V*_*k*,*m*_ is the number of voxels identified as activated in both the *k*th and the *m*th replications and *V_k_* and *V_m_* denote the number of voxels identified as activated in the *k*th and the *m*th experiments, respectively

$$\omega_{k,m}=\frac{2V_{k,m}}{V_{k}+V_{m}}$$ (1)

Therefore, ω_*k*,*m*_ is a ratio of the number of voxels identified as activated in both replications to the average number of voxels identified as activated in each replication. Note that this measure spans from 0 (i.e., no overlap) to 1 (perfect overlap) within the identified brain activation.

### Histology

#### TH and c-fos IHC

Perfused brains were fixated in 4.5% formalin (SAV Liquid Production GmbH, Flintsbach am Inn, Germany) and sectioned into three coronal parts (parts A, B, and C): One cut was performed approximately through the striatum, and the second one was performed through the SN. Then, the tissue was embedded in paraffin. Three rats from each group were selected on the basis of the previous fPET and fMRI results. For histology, 3- to 5-μm-thick sections were cut and stained with hematoxylin and eosin and correlated with the “Mouse Brain Atlas” (Allen Reference Atlas–Mouse Brain, available at https://atlas.brain-map.org/) to identify the sections containing the desired anatomical areas (striatum and SN). Adjacent to those sections, c-fos and TH IHC were performed on an automated immunostainer (Ventana Medical Systems Inc., Oro Valley, AZ, USA) according to the company’s protocols for open procedures with slight modifications. The slides were stained with the antibodies c-fos (SC-52, Santa Cruz Biotechnology, Dallas, TX, USA) and TH (#22941, Immunostar, Hudson, WI, USA). Appropriate positive and negative controls were used to confirm the adequacy of the staining. All samples were scanned with the Ventana DP200 (Roche, Basel, Switzerland) and processed with the Image Viewer MFC Application. Final image preparation was performed with Adobe Photoshop CS6.

The neuronal activation, revealed by c-fos IHC, was bilaterally quantified in the selected rats in the dorsal and ventral striatum and in the SN. For this, three ROIs were selected in each target region. See fig. S6 for more details on the selected ROIs. The number of positive and negative cells was counted at a magnification of ×400. A test for significant differences between right and left ROIs within the group and a comparison of the ROIs between GFP- and ChR2-expressing rats was performed using a Welch’s *t* test in Prism 9 (V. 9.3.1, GraphPad Software LLC, San Diego, CA, USA). No quantification of the TH IHC was performed.

#### GFP and YFP immunofluorescence staining

Adjacent to c-fos–, TH-, and hematoxylin and eosin–stained sections, a GFP/YFP staining was performed to control for AAV expression. Paraffin sections were rehydrated using a series of xylol and decreasing ethanol concentrations. Antigen retrieval was performed for 15 min at 95°C using universal antigen retrieval (R&D Systems Inc., Minneapolis, MN, USA). Sections were blocked in PBS containing 0.2% Triton X-100 and 5% bovine serum albumin and stained for GFP or YFP using an anti-GFP antibody (NB100-1614, Novus Biologicals, Biotechne, Wiesbaden Nordenstadt, Germany; 1:200) plus secondary anti-chicken Alexa Fluor 555 (A32932, Thermo Fisher Scientific Inc., Waltham, MA, USA; 1:200) together with 4′,6-diamidino-2-phenylindole (D1306, Thermo Fisher Scientific Inc., Waltham, MA, USA; 1:500). All antibodies were diluted in antibody diluent (IW-1000, IHC World LLC, Woodstock, MD, USA) and incubated for 1 hour at room temperature. Cover glasses were placed on top using antifade mounting medium (P36980, Thermo Fisher Scientific Inc., Waltham, MA, USA), and sections were acquired on a Leica DMi8 microscope interfaced with Leica LAS X software (Leica Microsystems CMS GmbH, Wetzlar, Germany). The images were further processed with ImageJ.

## Supplementary Materials

### This PDF file includes:

Supplementary Methods Figs. S1 to S7 Tables S1 to S6 References
