# Supplementary material for: Functional PET/MRI reveals active inhibition of neuronal activity during optogenetic activation of the nigrostriatal pathway
Source: Sci Adv. 2024 Oct 25;10(43):eadn2776. doi: 10.1126/sciadv.adn2776 (PMC11506239; doi:10.1126/sciadv.adn2776)
Supplement: Supplementary file 1 — Supplementary Methods Figs. S1 to S7 Tables S1 to S6 References [file sciadv.adn2776_sm.pdf]

Science Advances
AAAS

Supplementary Materials for

Functional PET/MRI reveals active inhibition of neuronal activity during optogenetic activation of the nigrostriatal pathway

Sabrina Haas *et al.*

Corresponding author: Kristina Herfert, Kristina.herfert@med.uni-tuebingen.de

Sci. Adv. **10**, eadn2776 (2024)
DOI: 10.1126/sciadv.adn2776

This PDF file includes:

Supplementary Methods
Figs. S1 to S7
Tables S1 to S6
References

Supplementary methods.

Multivariate pattern analysis (MVPA) applied to fMRI and fPET data.

Multivariate pattern analysis (MVPA) allows to evaluate differences between conditions with higher sensitivity than conventional univariate analysis by focusing on the analysis and comparison of distributed patterns of activity (*24, 25*). MVPA is frequently framed within the context of "brain decoding" applications, where distinct representational content can be discerned from fMRI activity patterns after performing a "training" or "learning phase". In this perspective, MVPA tools are commonly denoted as classifiers or, in broader terms, learning machines. Multivariate methods, including machine learning classifiers, prove to be highly sensitive tools for gauging brain information due to their ability to detect complex, high-dimensional mappings between spatially distributed patterns of brain activity and stimuli (*33*). The two datasets (fMRI and fPET) were pre-processed using the same sets of operations (see details in Data Preprocessing subsection). For the MVPA analyses, and following (*108*) we used normalized (i.e., transformation into a common reference space) and spatially smoothed data in order to reveal signatures or "biomarkers" of the optogenetic stimulation that would generalize across individuals. We applied whole-brain normalization to the fPET data to obtain 4D volumes that represented the dynamic relative [^{18}F]FDG uptake (time-activity) map (*17, 19, 96*). Prior to trial extraction, fPET and fMRI time courses were standardized using z normalization. As training exemplars for each class, response values were estimated for the individual optogenetic stimulation and baseline trials. The estimated trial response was calculated as the mean of three-minute time-span measurement points (i.e., duration equal to the length of the baseline blocks) around the peak BOLD or glucose uptake response during the optogenetic stimulation blocks relative to the correspondent pre-stimulus baseline activity. Using this strategy for both the fMRI and fPET datasets allowed us to obtain the same number of exemplars in each class, 12 per subject (6 per condition).

MVPA at the whole-brain level analysis using Spacenet classifiers and Searchlight.

We conducted whole-brain analysis employing SpaceNet classifiers sourced from the nilearn python package (*26*). These classifiers leverage spatial priors and regularization techniques to generate sparse yet structured coefficient maps. When the classifier achieves high out-of-sample classification accuracy, the resulting coefficient maps can be construed as information maps, indicating voxels wherein the collective activity carries discriminative information about the respective class (*109-111*). The SpaceNet classifiers were fit using 5-fold cross-validation and validated using a left-out test set (20%). For whole-brain SpaceNet MVPA analysis we employed a single train-test split approach to train and validate the model instead of cross-validation with permutation testing. Unfortunately, conducting permutation testing with SpaceNet would demand considerable computational resources, estimated at approximately 1500 hours per subject. Consequently, while SpaceNet yields interpretable maps, evaluating its null distribution presents a significant challenge.

Considering the above-mentioned limitations of the SpaceNet classifiers, we further performed a searchlight decoding analysis (*27*). This approach involves sliding a spherical window (of 1mm radius for our analysis) throughout the entire brain to map local multivariate effects and conducting independent decoding analyses within each sphere. We utilized the searchlight

implementation provided by nilearn (*26*), enabling us to acquire single-fold accuracy maps necessary to perform inference.

Classically, the decoding performance (used to measure the ability of the classifier to distinguish patterns associated with the different conditions included in the paradigm) is estimated separately in each participant. These within-subject measurements are then combined at the group level, to provide population-based inference, analogous to the standard hierarchical approach employed in univariate activation analyses. Since several limitations of this group-level strategy have been brought forward (*112-114*), we here used a different classifier-based framework to assess multivariate effects. This approach, referred to by distinct names such as across-subject classification, subject-transfer decoding or inter-subject pattern analysis (*115-119*), directly operates at the group-level by exploiting data from all available individuals in a single analysis at the group level: considering the data from all available individuals, the decoding performance is evaluated using data from new participants, namely those who were not part of the classifier's training dataset. When performing across-subject classification, a cross-validation of the type leave-one-subject-out enables a quantitative evaluation of the results that allows drawing inferences about the entire population from which the participant group was sampled, including individuals for which no data was available (*115, 119*). Across-subject classification exhibits enhanced sensitivity for detecting weak distributed effects and facilitates interpretation compared to standard hierarchical MVPA approaches, which often require further scrutiny due to potential ambiguities. Importantly, across-subject classification enables the identification of group-wise invariants within functional neuroimaging patterns, rendering it an invaluable tool for discerning neuromarkers or brain signatures, and offering a versatile framework for population-level multivariate analyses (*119*).

For our searchlight analysis we therefore employed a leave-one-subject-out cross-validation approach, in which the model accuracy was repeatedly computed on the data from the left-out subject. The approach trains on (n - 1) chunks, and classifies the remaining chunk, and repeats this for every chunk, also called fold. We created one chunk for each subject: 16 chunks for the fPET data and 18 chunks for the fMRI data.

To perform statistical inference at the group level, it is common practice to employ a t-test on decoding accuracies: this test evaluates whether the null hypothesis of chance-level average accuracy can be rejected, indicating the presence of a multivariate difference between conditions at the group level. However, various criticisms have been raised in the literature upon this approach, including concerns about the statistical distribution of classification accuracies (*112, 120*), the non-directional nature of identified group-information (*113*), and the potential bias introduced by confounding factors (*114*). Consequently, alternative methods have been developed [see, for example, (*112, 120-122*). Here, we employed a permutation test (Nichols & Holmes, 2002) to address the aforementioned limitations. This method evaluates the significance of the average accuracy at the group level in a non-parametric manner. Additionally, a permutation test allowed us to keep computational cost within reasonable bounds, a requirement that other alternatives, such as those suggested by (*122*) might not fulfill. We utilized the implementation provided by the SnPM toolbox (*28*) to analyze the accuracy maps from single-fold (for the inter-subject cross-validation), conducting 1000 permutations and applying a significance threshold of $p < 0.05$, corrected for family-wise-error (FWE).

MVPA ROI analysis.
For conducting MVPA ROI analysis, we performed an initial feature selection by considering only those voxels within the right SN. Estimated responses across relevant voxels from the right SN formed the feature vectors used to train the classifier. We evaluated a Gaussian Naive Bayes classifier (GNB) on the fMRI and fPET datasets. Analogous to the searchlight analysis, to assess multivariate effects, we used an across-subject classification approach. A positive result in this framework implies that the model has learnt an implicit rule from the training data that yields statistically significant generalization power on data from new subjects (*119*). We employed a leave-one-subject-out cross-validation for the MVPA ROI analysis. We tested the statistical significance of the accuracy of prediction using a permutation test: assuming there is no class information in the data, the labels can be permuted without altering the expected accuracy using a given classifier and number of features (i.e., this would equal chance level) (*28*). We performed 1000 permutations of a leave-one-subject-out cross-validated MVPA, and evaluated a GNB classifier with default parameters in Scikit-learn (*26*).

Functional and molecular connectivity analyses.
We used connectivity measures in order to attain a depiction of the optogenetic stimulation effects on neural dynamics in our experiment. We performed seed-based connectivity analysis, on both the fMRI and fPET datasets, to evaluate connectivity differences between optogenetic stimulation and baseline blocks (*29*).
The striatum receives axonal projections from SN dopaminergic neurons and houses a large population of GABAergic neurons. Consequently, we used the right striatum as seed region and assessed connectivity differences in response to the optogenetic stimulation.
Preprocessing of the fMRI and fPET data was performed as already described (see details in Data Preprocessing subsection) using SPM12 and the CONN toolbox (*123*), running in MATLAB (2019a, MathWorks, Inc.). The preprocessing steps included slice-timing correction, motion correction, normalization (i.e., transformation into a common reference space) and spatial smoothing. Following the procedure described in (*17, 19*) we applied whole-brain normalization to the fPET data, in order to obtain 4D volumes that represented the dynamic relative [^{18}F]FDG uptake (time-activity) map (*96*) A temporal high-pass filter with a cut-off frequency of 256 Hz was further applied to the fMRI data, with the purpose of removing scanner attributable low frequency drifts in the fMRI time series (*92*).

Seed-based connectivity analyses for fPET and fMRI data.
Functional connectivity depicts the temporal correlation of regional timeseries and is conceptualized to represent dynamic interaction and information sharing between brain regions (*29*), therefore characterizing spatially segregated functional networks at global scales. We calculated functional connectivity for the fMRI and fPET datasets using the CONN toolbox (*123*) in the form of seed-to-voxel analyses for assessing effects at the whole-brain level. Temporal correlations for the right striatum seed were computed for all voxels in the brain using a general linear model (GLM) for the contrast OGS > baseline. The functional connectivity analyses produced seed-to-voxel parameter estimate images, which were entered into population-level analyses. We report results thresholded at voxel level $p < 0.001$ (uncorrected) and cluster level $p < 0.05$ (family-wise error [FWE] corrected for multiple comparisons) for a valid voxel-wise inference approach (*95*).

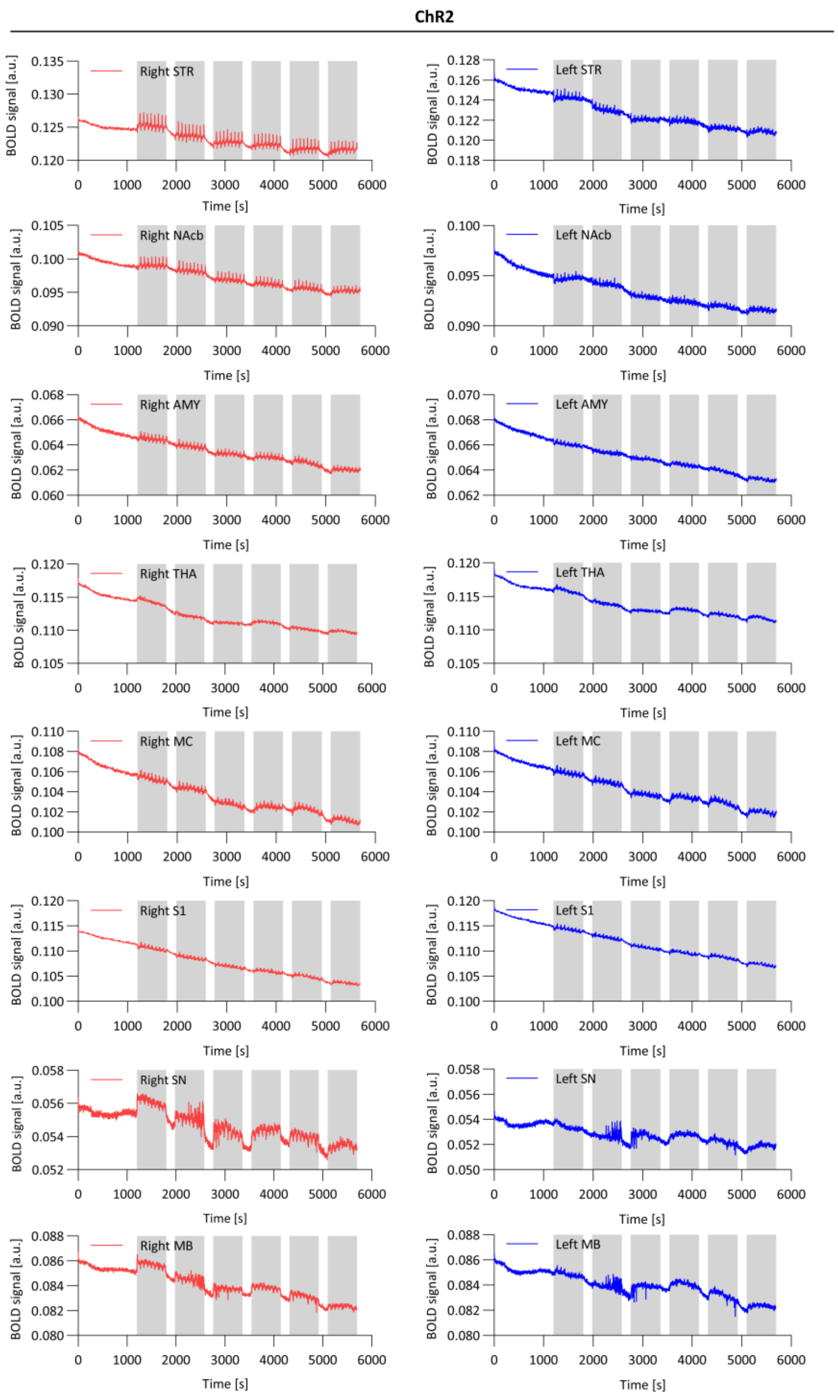

Fig. S1. BOLD-fMRI signal time courses of selected regions. Mean BOLD signal time courses over 95 minutes are shown for selected regions for ChR2 (n = 18) expressing rats. ChR2 expressing rats responded to stimulation (highlighted in grey) with positive BOLD signal changes in the right striatum, nucleus accumbens, amygdala, thalamus, midbrain and substantia nigra. Negative responses were obtained in the contralateral striatum, nucleus accumbens, amygdala and right and left cortical regions. Abbreviations: AMY, amygdala; MB, midbrain; MC, motor cortex; NAcb, nucleus accumbens; S1, somatosensory cortex; SN, substantia nigra; STR, striatum; THA, thalamus.

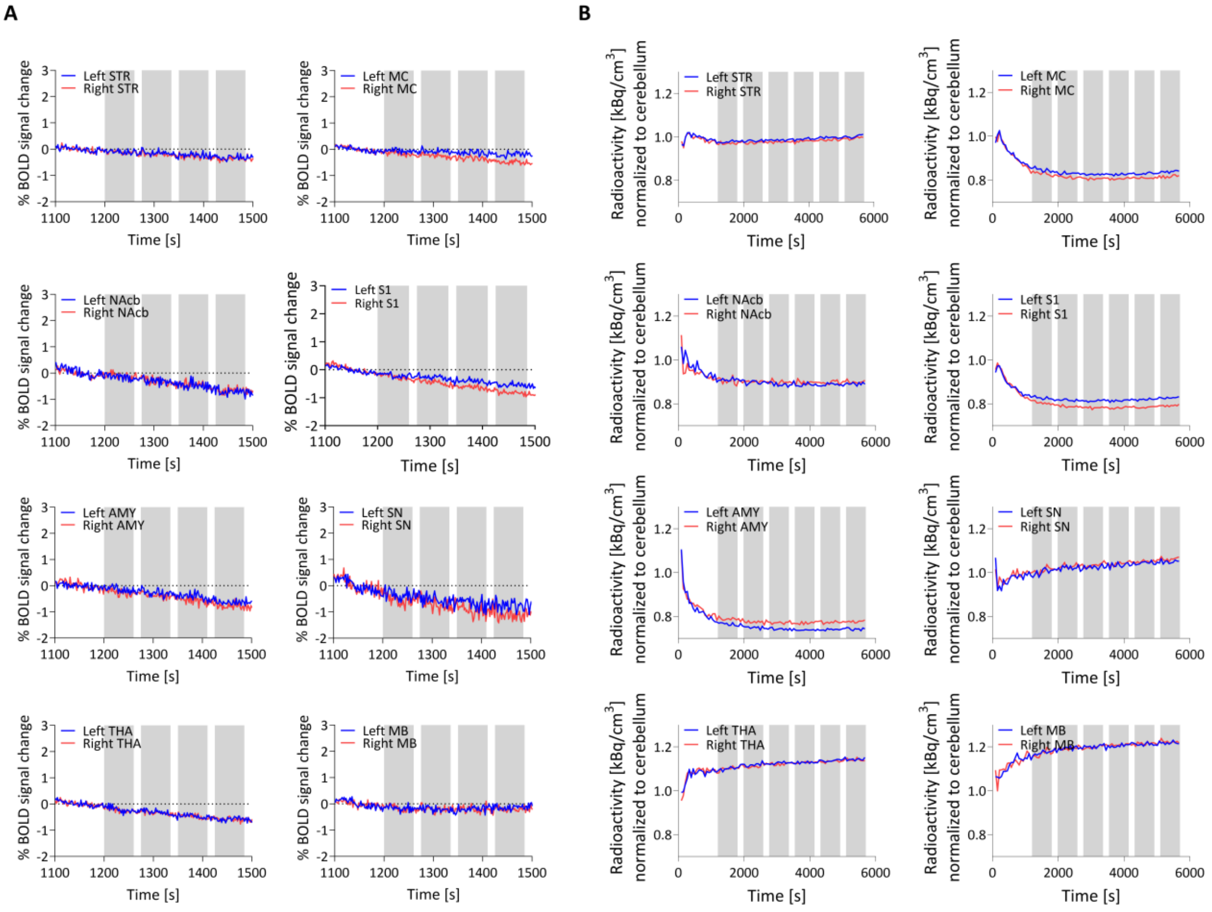

Fig. S2. %BOLD-fMRI signal changes and PET time activity curves in GFP rats. (**A**) Mean %BOLD signal changes over 400 seconds are shown for selected regions of GFP (n = 12) expressing rats. (**B**) Mean normalized [^{18}F]FDG time activity curves over 95 minutes are shown for selected regions of GFP (n = 14) expressing rats. In both modalities, GFP expressing rats did not respond to stimulation (highlighted in grey). Abbreviations: AMY, amygdala; MB, midbrain; MC, motor cortex; NAcb, nucleus accumbens; S1, somatosensory cortex; SN, substantia nigra; STR, striatum; THA, thalamus.

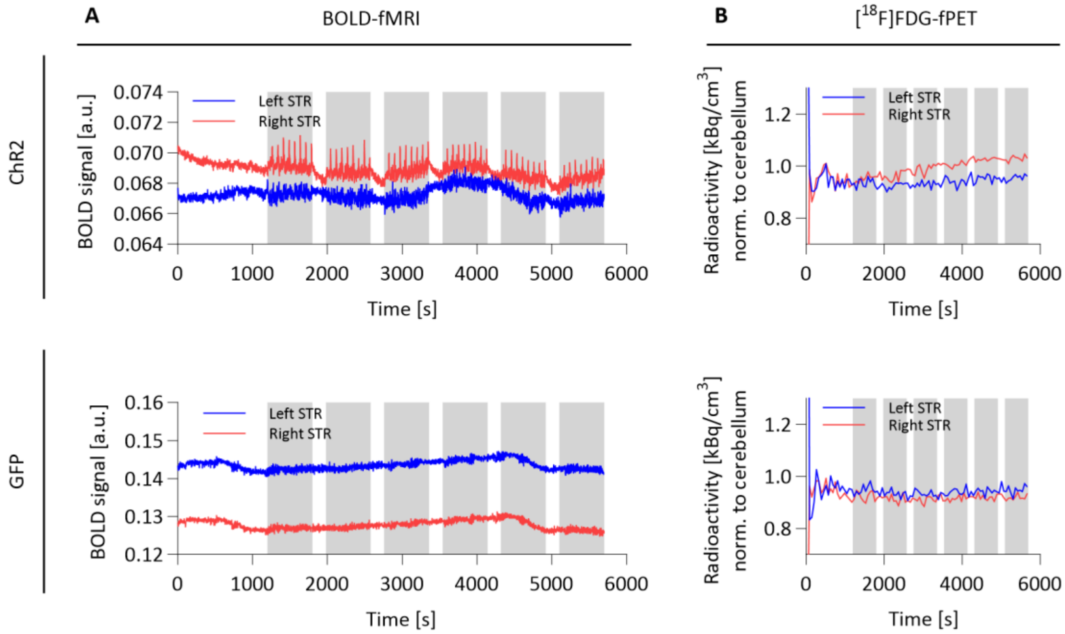

Fig. S3. [^{18}F]FDG-fPET and BOLD-fMRI activation on single animal level. (**A**) Mean BOLD signal time courses over 95 minutes are shown for the striatum for one exemplary ChR2 and GFP expressing rat. (**B**) Mean normalized time activity curves over 95 minutes are shown for the striatum of one exemplary ChR2 and GFP expressing rat. Grey bars indicate 10-minute stimulation blocks. Abbreviations: ChR2, channelrhodopsin-2; GFP, green fluorescent protein; STR, striatum.

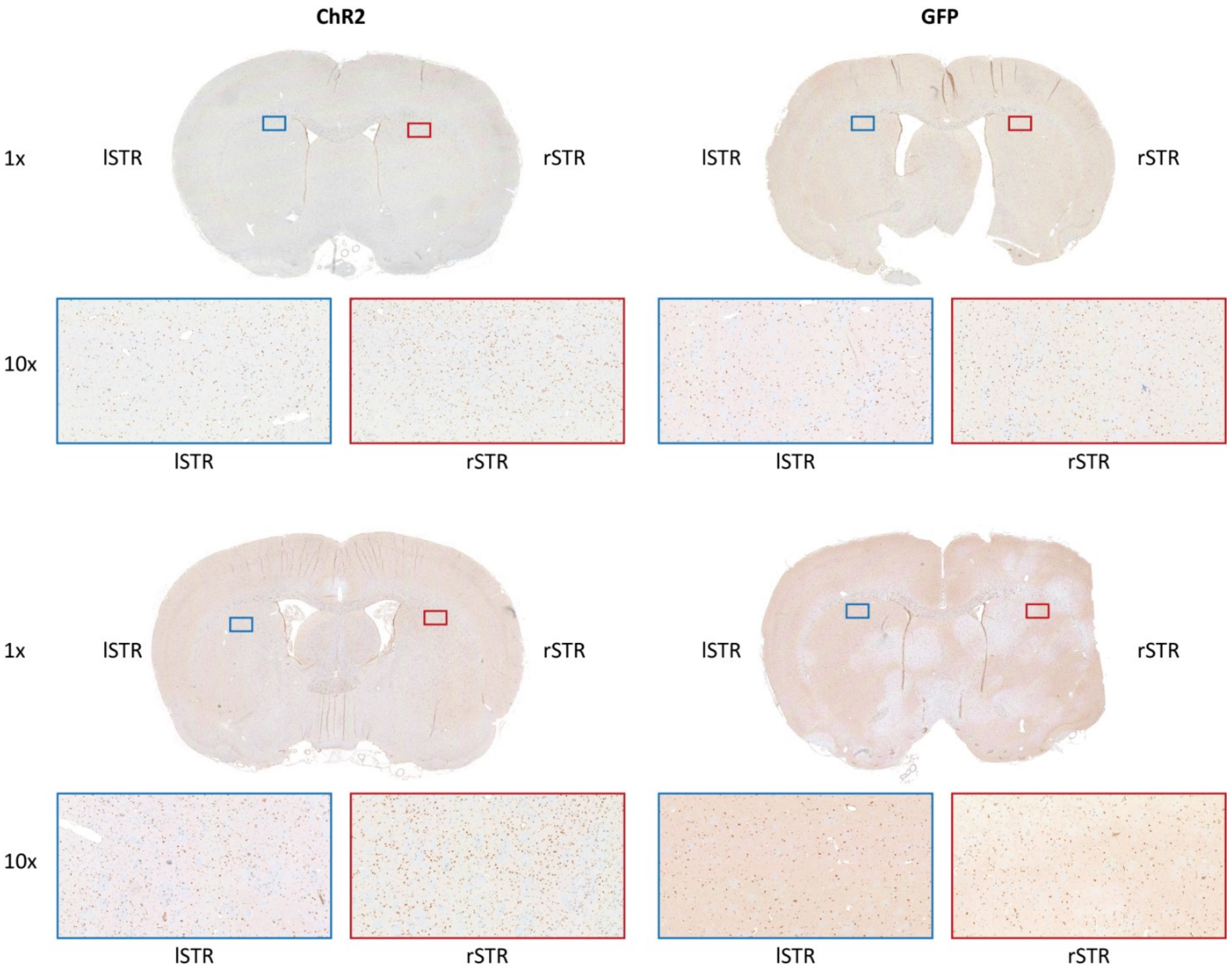

Fig. S4. c-fos immunohistochemical staining in the striatum. C-fos staining in the other two ChR2 and GFP rats not shown in Fig. 7 is shown for the dorsal striatum in 1× and 10× magnifications. A higher number of c-fos+ cells can be identified in the 10× magnification of the right striatum of the ChR2 rats. Abbreviations: ChR2, channelrhodopsin-2; GFP, green fluorescent protein; l, left; r, right; STR, striatum.

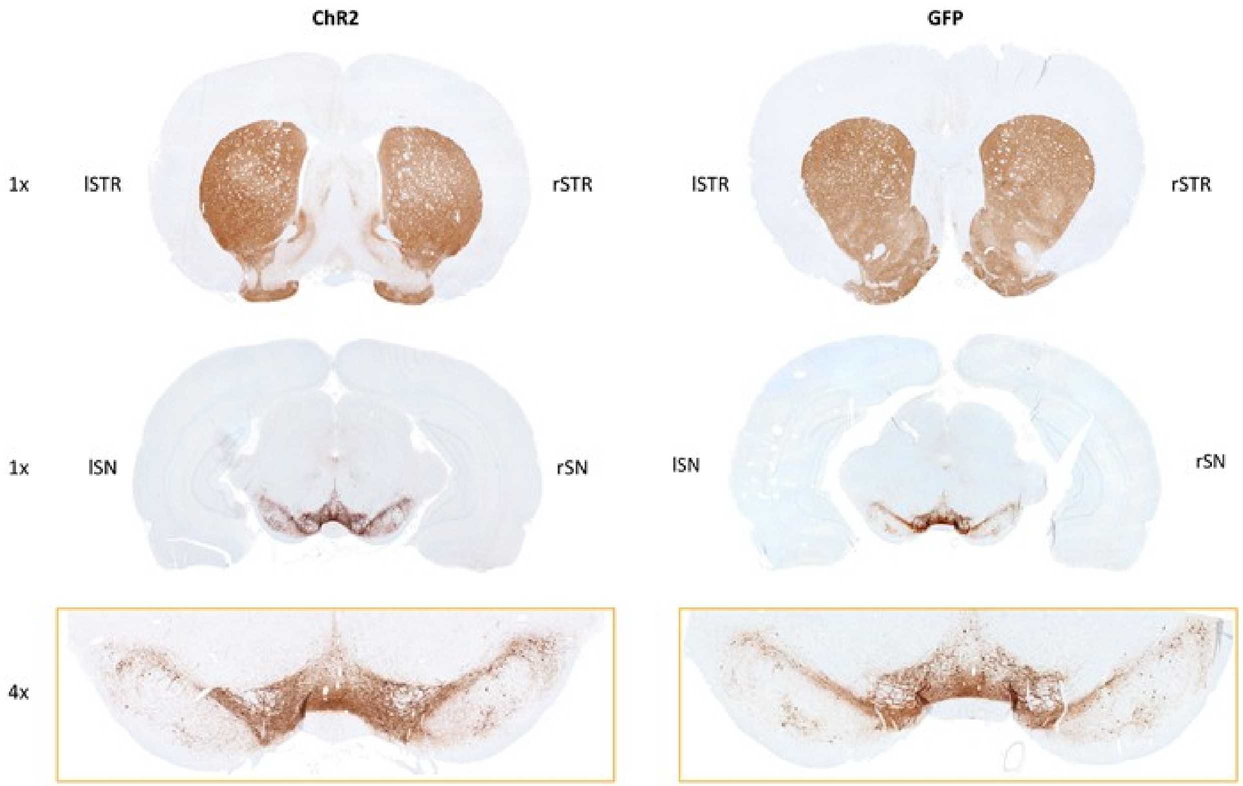

Fig. S5. Tyrosine hydroxylase immunohistochemical staining in the striatum and substantia nigra. Tyrosine hydroxylase staining in one exemplary ChR2 and one GFP rat is shown for the striatum in 1× and substantia nigra in 1× and 4× magnification. No qualitative left to right differences were identified in neither of the regions. Technical reasons are responsible for an uneven distribution of the staining. Abbreviations: ChR2, channelrhodopsin-2; GFP, green fluorescent protein; l, left; r, right; SN, substantia nigra; STR, striatum.

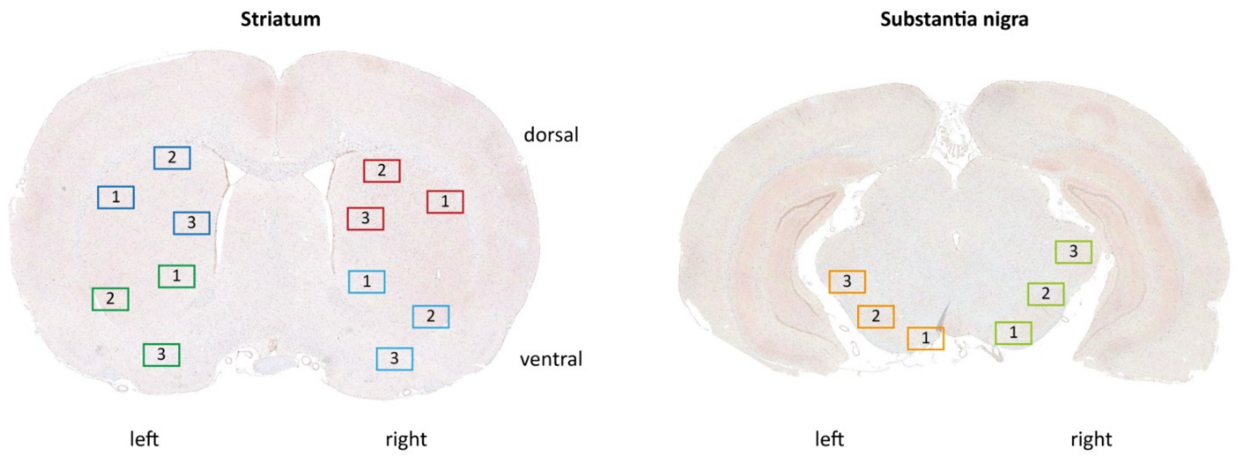

Fig. S6. Selected regions of interest for quantitative analysis of c-fos immunohistochemistry. Three regions of interest for c-fos quantification were drawn into the right and left, dorsal and ventral striatum and right and left substantia nigra of each selected rat.

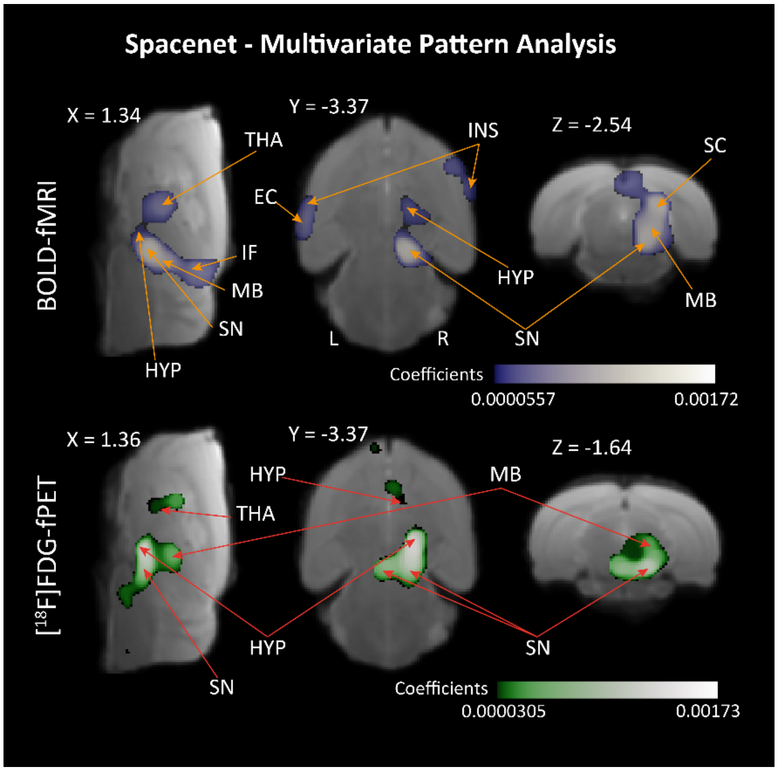

Fig. S7. Optogenetic stimulation coding at the whole-brain level using MVPA Spacenet classifiers. We trained multivariate classifiers to decode the optogenetic stimulation (vs. baseline) from patterns of hemodynamic responses or glucose uptake. The displayed results show group-level analysis of SpaceNet coefficient maps for the fMRI (top) and fPET (bottom) datasets, which can be interpreted as information maps specifying voxels whose joint signal changes contains information about the class (i.e., optogenetic stimulation). Abbreviations: AMY, amygdala; EC, entorhinal cortex; HYP, hypothalamus; INS, insular cortex; MB, midbrain; NAcb, nucleus accumbens; SC, superior colliculus; IC, inferior colliculus; SN, substantia nigra; STR, striatum; THA, thalamus; L, left; R, right.

Component	Kurtosis	Skeweness	Variability	Frequency
**ICA_9 (OGS)**	9.4702	1.5296	0.89649	0.0015126
**ICA_7**	6.1892	-1.1373	0.61559	0.0019337
**ICA_5**	6.0444	-0.029553	0.74643	0.0024379
**ICA_1**	5.9096	-0.92696	0.62911	0.0026374
**ICA_3**	5.5793	-1.6948	0.66834	0.0030253
**ICA_6**	5.1538	0.45623	0.71338	0.0029699
**ICA_4**	5.0307	0.20187	0.77642	0.002992
**ICA_2**	4.9093	0.3029	0.77882	0.0029532
**ICA_11**	4.8252	0.46421	0.81305	0.0027648
**ICA_12**	4.7691	0.42947	0.74488	0.0031804
**ICA_18**	4.5608	-0.071605	0.77426	0.0026208
**ICA_16**	4.1498	0.35611	0.92187	0.0031472
**ICA_10**	3.7893	0.17116	0.85876	0.0032524
**ICA_13**	3.4033	0.043359	0.86731	0.0030973
**ICA_14**	2.8282	0.045367	1.011	0.0031305
**ICA_8**	2.7453	0.034452	0.87565	0.0030862
**ICA_20**	2.6707	-0.013394	0.77546	0.0034574
**ICA_15**	2.5996	0.008139	0.80949	0.0029699
**ICA_17**	2.5655	-0.029454	0.77274	0.0032912
**ICA_19**	2.226	0.12018	0.79925	0.0028092

Table S1. Independent component analysis (ICA). Descriptive measures derived from the independent component's voxels values distribution for all 20 components (kurtosis sorted).

Brain region (ROI)	Distance activation centers [mm] BETWEEN	Distance activation centers [mm] WITHIN	Dice similarity coefficient BETWEEN	Dice similarity coefficient WITHIN
**R AMY**	5.0	5.0	-	-
**R HIP posterior**	2.2	-	-	-
**R HYP**	1.6	2.1	0.277	0.018
**R MB**	2.1	2.7	0.165	0
**PAG**	1.9	1.0	-	-
**R SN**	1.3	0.9	0.026	0
**R S1**	-	2.8	-	0.042
**R INS**	-	3.7	-	0.080
**R NAcb**		0.9	-	0.444
**R STR**	1.2	2.9	0.548	0.743
**R THA**	3.5	-	0.024	0.0221

Table S2. Distance of t-value peak location between fPET and fMRI and Dice similarity coefficient (between- and within-group analysis). Abbreviations: AMY, amygdala; HIP, hippocampus; HYP, hypothalamus; ICA, independent component analysis; L, left; MB, midbrain; PAG, periaqueductal gray; R, right; SN, substantia nigra; STR, striatum; THA, thalamus; NAcb, nucleus accumbens; INS, insular cortex; S1, somatosensory cortex.

	ChR2		GFP	
**Brain region (ROI)**	**Right**	**Left**	**Right**	**Left**
**Dorsal striatum**	73 ± 4.0%	48 ± 5.6%	47 ± 6.7%	46 ± 2.5%
**Ventral striatum**	50 ± 17%	42 ± 11%	44 ± 11%	45 ± 15%
**Substantia nigra**	87 ± 4.0%	82 ± 5.5%	82 ± 5.7%	86 ± 4.2%

Table S3. Percentage of c-fos+ cells. Abbreviations: ChR2, channelrhodopsin-2; GFP, green fluorescent protein; ROI, region of interest.

Brain region (ROI)	ROI volume [mm³]	# voxels	Abbreviation
**R/ L nucleus accumbens**	7.9	993	NAcb
**R/ L amygdala**	21.1	2640	AMY
**R/ L caudate putamen**	43.5	5444	STR
**R/ L auditory cortex**	27.5	3440	AC
**R/ L cingulate cortex**	14.5	1810	Cg
**R/ L entorhinal cortex**	59.0	7377	EC
**R/ L insular cortex**	21.1	2641	INS
**R/ L medial prefrontal cortex**	6.3	788	mPFC
**R/ L motor cortex**	32.6	4076	MC
**R/ L orbitofrontal cortex**	18.9	2367	OFC
**R/ L parietal cortex**	7.6	954	PaC
**R/ L retrosplenial cortex**	18.9	2365	RS
**R/ L somatosensory cortex**	71.6	8950	S1
**R/ L visual cortex**	36.1	4517	V1
**R/ L anterodorsal hippocampus**	25.1	3133	HIP ant.
**R/ L posterior hippocampus**	9.8	1223	HIP post.
**R/ L hypothalamus**	18.4	2294	HYP
**R/ L olfactory cortex**	14.0	1751	OC
**R/ L superior colliculus**	7.1	892	SC
**R/ L midbrain**	11.4	1431	MB
**R/ L ventral tegmental area/ substantia nigra**	5.5	691	SN
**R/ L cerebellum – grey matter**	75.0	9374	CG
**R/ L cerebellum – white matter**	23.4	2938	CW
**R/ L inferior colliculus**	5.7	718	IC
**R/ L thalamus**	30.7	3839	THA
**medulla**	58.2	2944	Med
**periaqueductal gray**	9.9	1238	PAG
**pituitary gland**	5.8	733	PG
**septum**	9.4	1170	Sep

Table S4. Characteristics and abbreviations of selected regions of interest (Schiffer rat brain atlas)
Abbreviations: L, left; R, right; ROI, region of interest.

Region	Cluster Peak	Voxels	Max t-value (z-value)	Mean t (std.)	*p*-value (FWE)
**Seed-based *molecular* connectivity (SBC)**					
Midbrain R*	0.55 -2.8 -1.2	581	10.38 (5.54)	5.04 (0.99)	< 0.0001
Substantia nigra R*		510		5.22 (1.22)	cl.
Hypothalamus R		200		4.46 (0.55)	cl.
Substantia nigra L		10		3.99 (0.22)	cl.
Thalamus R		10		3.88 (0.15)	cl.
**Seed-based *functional* connectivity (SBC)**					
Striatum R*	4.52 -3.2 3.6	4949	18.81 (7.16)	8.11 (2.39)	< 0.0001
Amygdala R*		739		6.97 (1.74)	cl.
Nucleus Accumbens R*		586		6.45 (1.01)	cl.

Table S5. Functional and molecular connectivity. Results are shown at threshold $p < 0.001$ voxel-level uncorrected, $p < 0.05$ cluster-level FWE-corrected; * markings show brain regions with significant signal changes at voxel-level FWE-corrected $p < 0.05$). Results of group seed-based connectivity (SBC) analysis, with the right striatum as seed region for the contrast optogenetic stimulation vs. baseline. The regions described showed stronger positive functional/metabolic connectivity with the seed region during the optogenetic stimulation compared to baseline. Abbreviations: L, left; R, right; Cl., areas integrating the above detailed cluster-level p-value.

Region	Coordinates	Voxels	Mean t (std.)
**fMRI - Searchlight**			
Striatum R	5.4 -1.6 2	4442	5.48 (1.07)
Thalamus R	1.22 -2.8 1.6	1399	4.92 (0.89)
Midbrain R	0.55 -2.6 -2.8	1027	5.54 (1.37)
Substantia nigra R	0.11 -2.8 -1.6	512	4.85 (0.91)
Hypothalamus R	2.1 -3.2 4	320	4.49 (0.69)
Nucleus accumbens R	1.88 -2.4 7	123	6.61 (0.91)
Insular cortex R	6.28 -2 2.2	97	4.96 (1.01)
Substantia nigra L	-0.54 -2.8 -1.6	76	5.82 (1.59)
Amygdala R	5.4 -3.4 2.2	44	5.38 (1.07)
Hypothalamus L	-2.62 -4.47 1.61	41	4.59 (0.74)
Thalamus L	-0.97 -1.11 3.19	38	4.11 (0.43)
Nucleus accumbens L	-1.49 -3.37 6.18	27	4.76 (0.89)
**fPET - Searchlight**			
Hypothalamus R	0.11 -4 -1.4	740	5.07 (0.91)
Thalamus R	2.54 -2 0.99	671	5.16 (1.18)
Substantia nigra R	0.99 -3.6 -1.4	565	5.35 (1.11)
Midbrain R	2.1 -3.2 -2.8	425	4.84 (0.74)
Striatum R	2.54 -1.6 4.8	173	4.16 (0.40)
Substantia nigra L	-0.55 -2.8 -1.5	78	5.38 (0.70)
Amygdala R	3.42 -3.8 -1.2	20	4.22 (0.54)
Amygdala L	-3.56 -5.30 2.16	19	4.69 (0.95)
Hypothalamus L	-0.32 -3.2 0.99	18	4.38 (0.75)
Nucleus accumbens L		11	4.21 (0.33)
Thalamus L	-0.98 -2.4 -6.55	8	4.13 (0.30)

Table S6. Searchlight analysis. Regions where searchlight-based classification analysis discriminates between optogenetic stimulation vs. baseline trials (searchlight classification >60%, whole-brain cluster-corrected $p < 0.05$ via comparison with 1000 random permutations). Coordinates denote the 3D center of gravity of each region. Abbreviations: L, left; R, right.

REFERENCES AND NOTES

1. B. Liss, J. Roeper, Individual dopamine midbrain neurons: Functional diversity and flexibility in health and disease. *Brain Res. Rev.* **58**, 314–321 (2008).

2. S. D. Jamadar, P. G. D. Ward, T. G. Close, A. Fornito, M. Premaratne, K. O'Brien, D. Stäb, Z. Chen, N. J. Shah, G. F. Egan, Simultaneous BOLD-fMRI and constant infusion FDG-PET data of the resting human brain. *Sci. Data* **7**, 363 (2020).

3. C. Y. Sander, H. D. Hansen, H. Y. Wey, Advances in simultaneous PET/MR for imaging neuroreceptor function. *J. Cereb. Blood Flow Metab.* **40**, 1148–1166 (2020).

4. A. Hahn, M. Breakspear, L. Rischka, W. Wadsak, G. M. Godbersen, V. Pichler, P. Michenthaler, T. Vanicek, M. Hacker, S. Kasper, R. Lanzenberger, L. Cocchi, Reconfiguration of functional brain networks and metabolic cost converge during task performance. *eLife* **9**, e52443 (2020).

5. T. M. Ionescu, M. Amend, T. Watabe, J. Hatazawa, A. Maurer, G. Reischl, B. J. Pichler, H. F. Wehrl, K. Herfert, Neurovascular uncoupling: Multimodal imaging delineates the acute effects of 3,4-methylenedioxymethamphetamine. *J. Nucl. Med.* **64**, 466–471 (2023).

6. T. M. Ionescu, M. Amend, R. Hafiz, B. B. Biswal, A. Maurer, B. J. Pichler, H. F. Wehrl, K. Herfert, Striatal and prefrontal D2R and SERT distributions contrastingly correlate with default-mode connectivity. *Neuroimage* **243**, 118501 (2021).

7. S. Marciano, T. M. Ionescu, R. S. Saw, R. Y. Cheong, D. Kirik, A. Maurer, B. J. Pichler, K. Herfert, Combining CRISPR-Cas9 and brain imaging to study the link from genes to molecules to networks. *Proc. Natl. Acad. Sci. U.S.A.* **119**, e2122552119 (2022).

8. K. Fischer, V. Sossi, A. von Ameln-Mayerhofer, G. Reischl, B. J. Pichler, Quantification of dopamine transporters in mice with unilateral 6-OHDA lesions using [^{11}C]methylphenidate and PET. *Neuroimage* **59**, 2413–2422 (2012).

9. K. Fischer, V. Sossi, A. Schmid, M. Thunemann, F. C. Maier, M. S. Judenhofer, J. G. Mannheim, G. Reischl, B. J. Pichler, Noninvasive nuclear imaging enables the in vivo

quantification of striatal dopamine receptor expression and raclopride affinity in mice. *J. Nucl. Med.* **52**, 1133–1141 (2011).

10. N. Y. Lettfuss, K. Fischer, V. Sossi, B. J. Pichler, A. von Ameln-Mayerhofer, Imaging DA release in a rat model of L-DOPA-induced dyskinesias: A longitudinal in vivo PET investigation of the antidyskinetic effect of MDMA. *Neuroimage* **63**, 423–433 (2012).

11. L. J. Stiernman, F. Grill, A. Hahn, L. Rischka, R. Lanzenberger, V. Panes Lundmark, K. Riklund, J. Axelsson, A. Rieckmann, Dissociations between glucose metabolism and blood oxygenation in the human default mode network revealed by simultaneous PET-fMRI. *Proc. Natl. Acad. Sci. U.S.A.* **118**, e2021913118 (2021).

12. E. S. Boyden, F. Zhang, E. Bamberg, G. Nagel, K. Deisseroth, Millisecond-timescale, genetically targeted optical control of neural activity. *Nat. Neurosci.* **8**, 1263–1268 (2005).

13. M. Benoit-Marand, E. Borrelli, F. Gonon, Inhibition of dopamine release via presynaptic D2 receptors: Time course and functional characteristics in vivo. *J. Neurosci.* **21**, 9134–9141 (2001).

14. M. Villien, H.-Y. Wey, J. B. Mandeville, C. Catana, J. R. Polimeni, C. Y. Sander, N. R. Zürcher, D. B. Chonde, J. S. Fowler, B. R. Rosen, J. M. Hooker, Dynamic functional imaging of brain glucose utilization using fPET-FDG. *Neuroimage* **100**, 192–199 (2014).

15. H. F. Wehrl, M. Hossain, K. Lankes, C. C. Liu, I. Bezrukov, P. Martirosian, F. Schick, G. Reischl, B. J. Pichler, Simultaneous PET-MRI reveals brain function in activated and resting state on metabolic, hemodynamic and multiple temporal scales. *Nat. Med.* **19**, 1184–1189 (2013).

16. A. Hahn, G. Gryglewski, L. Nics, M. Hienert, L. Rischka, C. Vraka, H. Sigurdardottir, T. Vanicek, G. M. James, R. Seiger, A. Kautzky, L. Silberbauer, W. Wadsak, M. Mitterhauser, M. Hacker, S. Kasper, R. Lanzenberger, Quantification of task-specific glucose metabolism with constant infusion of ^{18}F-FDG. *J. Nucl. Med.* **57**, 1933–1940 (2016).

17. S. D. Jamadar, P. G. D. Ward, S. Li, F. Sforazzini, J. Baran, Z. Chen, G. F. Egan, Simultaneous task-based BOLD-fMRI and [18-F] FDG functional PET for measurement of neuronal metabolism in the human visual cortex. *Neuroimage* **189**, 258–266 (2019).

18. L. Rischka, G. Gryglewski, S. Pfaff, T. Vanicek, M. Hienert, M. Klobl, M. Hartenbach, A. Haug, W. Wadsak, M. Mitterhauser, M. Hacker, S. Kasper, R. Lanzenberger, A. Hahn, Reduced task durations in functional PET imaging with [^{18}F]FDG approaching that of functional MRI. *Neuroimage* **181**, 323–330 (2018).

19. S. Li, S. D. Jamadar, P. G. D. Ward, M. Premaratne, G. F. Egan, Z. Chen, Analysis of continuous infusion functional PET (fPET) in the human brain. *Neuroimage* **213**, 116720 (2020).

20. F. De Martino, F. Gentile, F. Esposito, M. Balsi, F. Di Salle, R. Goebel, E. Formisano, Classification of fMRI independent components using IC-fingerprints and support vector machine classifiers. *Neuroimage* **34**, 177–194 (2007).

21. E. Formisano, F. Esposito, N. Kriegeskorte, G. Tedeschi, F. Di Salle, R. Goebel, Spatial independent component analysis of functional magnetic resonance imaging time-series: Characterization of the cortical components. *Neurocomputing* **49**, 241–254 (2002).

22. W. Lu, J. C. Rajapakse, Eliminating indeterminacy in ICA. *Neurocomputing* **50**, 271–290 (2003).

23. C. H. Moritz, B. P. Rogers, M. E. Meyerand, Power spectrum ranked independent component analysis of a periodic fMRI complex motor paradigm. *Hum. Brain Mapp.* **18**, 111–122 (2003).

24. T. Davis, K. F. LaRocque, J. A. Mumford, K. A. Norman, A. D. Wagner, R. A. Poldrack, What do differences between multi-voxel and univariate analysis mean? How subject-, voxel-, and trial-level variance impact fMRI analysis. *Neuroimage* **97**, 271–283 (2014).

25. F. Pereira, T. Mitchell, M. Botvinick, Machine learning classifiers and fMRI: A tutorial overview. *Neuroimage* **45**, S199–S209 (2009).

26. A. Abraham, F. Pedregosa, M. Eickenberg, P. Gervais, A. Mueller, J. Kossaifi, A. Gramfort, B. Thirion, G. Varoquaux, Machine learning for neuroimaging with scikit-learn. *Front. Neuroinform.* **8**, 14 (2014).

27. N. Kriegeskorte, R. Goebel, P. Bandettini, Information-based functional brain mapping. *Proc. Natl. Acad. Sci.* **103**, 3863–3868 (2006).

28. T. E. Nichols, A. P. Holmes, Nonparametric permutation tests for functional neuroimaging: A primer with examples. *Hum. Brain Mapp.* **15**, 1–25 (2002).

29. K. J. Friston, Functional and effective connectivity: A review. *Brain Connect.* **1**, 13–36 (2011).

30. N. X. Tritsch, J. B. Ding, B. L. Sabatini, Dopaminergic neurons inhibit striatal output through non-canonical release of GABA. *Nature* **490**, 262–266 (2012).

31. N. X. Tritsch, W. J. Oh, C. Gu, B. L. Sabatini, Midbrain dopamine neurons sustain inhibitory transmission using plasma membrane uptake of GABA, not synthesis. *eLife* **3**, e01936 (2014).

32. J. C. Patel, A. D. Sherpa, R. Melani, P. Witkovsky, M. R. Wiseman, B. O'Neill, C. Aoki, N. X. Tritsch, M. E. Rice, GABA co-released from striatal dopamine axons dampens phasic dopamine release through autoregulatory $GABA_A$ receptors. *Cell Rep.* **43**, 113834 (2024).

33. T. M. Mitchell, R. Hutchinson, R. S. Niculescu, F. Pereira, X. Wang, M. Just, S. Newman, Learning to decode cognitive states from brain images. *Mach. Learn.* **57**, 145–175 (2004).

34. D. Sulzer, S. J. Cragg, M. E. Rice, Striatal dopamine neurotransmission: Regulation of release and uptake. *Basal Ganglia* **6**, 123–148 (2016).

35. C. P. Ford, The role of D2-autoreceptors in regulating dopamine neuron activity and transmission. *Neuroscience* **282**, 13–22 (2014).

36. A. Andrea, E. L.-O. José, R. Maria, M. Claudia De, F. W. Hopf, I. Ciro, H. Briac, J. Jacob, K. Chisato, W. Marc, G. C. Marc, B. Antonello, S. David, B. Emiliana, Dual control of

dopamine synthesis and release by presynaptic and postsynaptic dopamine D2 receptors. *J. Neurosci.* **32**, 9023 (2012).

37. A. B. Nelson, N. Hammack, C. F. Yang, N. M. Shah, R. P. Seal, A. C. Kreitzer, Striatal cholinergic interneurons drive GABA release from dopamine terminals. *Neuron* **82**, 63–70 (2014).

38. T. Koos, J. M. Tepper, Inhibitory control of neostriatal projection neurons by GABAergic interneurons. *Nat. Neurosci.* **2**, 467–472 (1999).

39. J. W. Mink, The basal ganglia: Focused selection and inhibition of competing motor programs. *Prog. Neurobiol.* **50**, 381–425 (1996).

40. C. R. Gerfen, T. M. Engber, L. C. Mahan, Z. Susel, T. N. Chase, F. J. Monsma Jr., D. R. Sibley, D1 and D2 dopamine receptor-regulated gene expression of striatonigral and striatopallidal neurons. *Science* **250**, 1429–1432 (1990).

41. D. J. Surmeier, J. Ding, M. Day, Z. Wang, W. Shen, D1 and D2 dopamine-receptor modulation of striatal glutamatergic signaling in striatal medium spiny neurons. *Trends Neurosci.* **30**, 228–235 (2007).

42. R. L. Albin, A. B. Young, J. B. Penney, The functional anatomy of basal ganglia disorders. *Trends Neurosci.* **12**, 366–375 (1989).

43. M. R. DeLong, Primate models of movement disorders of basal ganglia origin. *Trends Neurosci.* **13**, 281–285 (1990).

44. A. V. Kravitz, B. S. Freeze, P. R. Parker, K. Kay, M. T. Thwin, K. Deisseroth, A. C. Kreitzer, Regulation of parkinsonian motor behaviours by optogenetic control of basal ganglia circuitry. *Nature* **466**, 622–626 (2010).

45. G. Cui, S. B. Jun, X. Jin, M. D. Pham, S. S. Vogel, D. M. Lovinger, R. M. Costa, Concurrent activation of striatal direct and indirect pathways during action initiation. *Nature* **494**, 238–242 (2013).

46. O. Hikosaka, Y. Takikawa, R. Kawagoe, Role of the basal ganglia in the control of purposive saccadic eye movements. *Physiol. Rev.* **80**, 953–978 (2000).

47. J. F. Keeler, D. O. Pretsell, T. W. Robbins, Functional implications of dopamine D1 vs. D2 receptors: A 'prepare and select' model of the striatal direct vs. indirect pathways. *Neuroscience* **282**, 156–175 (2014).

48. P. Calabresi, B. Picconi, A. Tozzi, V. Ghiglieri, M. Di Filippo, Direct and indirect pathways of basal ganglia: A critical reappraisal. *Nat. Neurosci.* **17**, 1022–1030 (2014).

49. E. K. Richfield, J. B. Penney, A. B. Young, Anatomical and affinity state comparisons between dopamine D1 and D2 receptors in the rat central nervous system. *Neuroscience* **30**, 767–777 (1989).

50. J. K. Dreyer, K. F. Herrik, R. W. Berg, J. D. Hounsgaard, Influence of phasic and tonic dopamine release on receptor activation. *J. Neurosci.* **30**, 14273–14283 (2010).

51. J. B. Mandeville, C. Y. M. Sander, B. G. Jenkins, J. M. Hooker, C. Catana, W. Vanduffel, N. M. Alpert, B. R. Rosen, M. D. Normandin, A receptor-based model for dopamine-induced fMRI signal. *Neuroimage* **75**, 46–57 (2013).

52. C. Y. Sander, J. M. Hooker, C. Catana, M. D. Normandin, N. M. Alpert, G. M. Knudsen, W. Vanduffel, B. R. Rosen, J. B. Mandeville, Neurovascular coupling to D2/D3 dopamine receptor occupancy using simultaneous PET/functional MRI. *Proc. Natl. Acad. Sci. U.S.A.* **110**, 11169–11174 (2013).

53. L. Hunger, A. Kumar, R. Schmidt, Abundance compensates kinetics: Similar effect of dopamine signals on D1 and D2 receptor populations. *J. Neurosci.* **40**, 2868–2881 (2020).

54. P. F. Marcott, A. A. Mamaligas, C. P. Ford, Phasic dopamine release drives rapid activation of striatal D2-receptors. *Neuron* **84**, 164–176 (2014).

55. C. Yapo, A. G. Nair, L. Clement, L. R. Castro, J. Hellgren Kotaleski, P. Vincent, Detection of phasic dopamine by D1 and D2 striatal medium spiny neurons. *J. Physiol.* **595**, 7451–7475 (2017).

56. S. Navailles, P. De Deurwaerdere, Imbalanced dopaminergic transmission mediated by serotonergic neurons in L-DOPA-induced dyskinesia. *Parkinsons Dis.* **2012**, 323686 (2012).

57. R. C. Sellnow, J. H. Newman, N. Chambers, A. R. West, K. Steece-Collier, I. M. Sandoval, M. J. Benskey, C. Bishop, F. P. Manfredsson, Regulation of dopamine neurotransmission from serotonergic neurons by ectopic expression of the dopamine D2 autoreceptor blocks levodopa-induced dyskinesia. *Acta Neuropathol. Commun.* **7**, 8 (2019).

58. G. M. Godbersen, S. Klug, W. Wadsak, V. Pichler, J. Raitanen, A. Rieckmann, L. Stiernman, L. Cocchi, M. Breakspear, M. Hacker, R. Lanzenberger, A. Hahn, Task-evoked metabolic demands of the posteromedial default mode network are shaped by dorsal attention and frontoparietal control networks. *eLife* **12**, e84683 (2023).

59. I. Vanzetta, A. Grinvald, Increased cortical oxidative metabolism due to sensory stimulation: Implications for functional brain imaging. *Science* **286**, 1555–1558 (1999).

60. D. Malonek, U. Dirnagl, U. Lindauer, K. Yamada, I. Kanno, A. Grinvald, Vascular imprints of neuronal activity: Relationships between the dynamics of cortical blood flow, oxygenation, and volume changes following sensory stimulation. *Proc. Natl. Acad. Sci. U.S.A.* **94**, 14826–14831 (1997).

61. T. A. Woolsey, C. M. Rovainen, S. B. Cox, M. H. Henegar, G. E. Liang, D. Liu, Y. E. Moskalenko, J. Sui, L. Wei, Neuronal units linked to microvascular modules in cerebral cortex: Response elements for imaging the brain. *Cereb. Cortex* **6**, 647–660 (1996).

62. N. Harel, S. P. Lee, T. Nagaoka, D. S. Kim, S. G. Kim, Origin of negative blood oxygenation level-dependent fMRI signals. *J. Cereb. Blood Flow Metab.* **22**, 908–917 (2002).

63. Y. Liu, H. Shen, Z. Zhou, D. Hu, Sustained negative BOLD response in human fMRI finger tapping task. *PLOS ONE* **6**, e23839 (2011).

64. A. Rodriguez-Baeza, F. Reina-De La Torre, M. Ortega-Sanchez, J. Sahuquillo-Barris, Perivascular structures in corrosion casts of the human central nervous system: A confocal laser and scanning electron microscope study. *Anat. Rec.* **252**, 176–184 (1998).

65. M. Moraschi, M. DiNuzzo, F. Giove, On the origin of sustained negative BOLD response. *J. Neurophysiol.* **108**, 2339–2342 (2012).

66. Y. Y. Shih, C. C. Chen, B. C. Shyu, Z. J. Lin, Y. C. Chiang, F. S. Jaw, Y. Y. Chen, C. Chang, A new scenario for negative functional magnetic resonance imaging signals: Endogenous neurotransmission. *J. Neurosci.* **29**, 3036–3044 (2009).

67. L. Edvinsson, J. McCulloch, J. Sharkey, Vasomotor responses of cerebral arterioles in situ to putative dopamine receptor agonists. *Br. J. Pharmacol.* **85**, 403–410 (1985).

68. D. H. Cerri, D. L. Albaugh, L. R. Walton, B. Katz, T. W. Wang, T. H. Chao, W. Zhang, R. J. Nonneman, J. Jiang, S. H. Lee, A. Etkin, C. N. Hall, G. D. Stuber, Y. I. Shih, Distinct neurochemical influences on fMRI response polarity in the striatum. *Nat. Commun.* **15**, 1916 (2024).

69. X. Yu, D. Glen, S. Wang, S. Dodd, Y. Hirano, Z. Saad, R. Reynolds, A. C. Silva, A. P. Koretsky, Direct imaging of macrovascular and microvascular contributions to BOLD fMRI in layers IV-V of the rat whisker-barrel cortex. *Neuroimage* **59**, 1451–1460 (2012).

70. N. Sirmpilatze, J. Baudewig, S. Boretius, Temporal stability of fMRI in medetomidine-anesthetized rats. *Sci. Rep.* **9**, 16673 (2019).

71. J. Grandjean, G. Desrosiers-Gregoire, C. Anckaerts, D. Angeles-Valdez, F. Ayad, D. A. Barrière, I. Blockx, A. B. Bortel, M. Broadwater, B. M. Cardoso, M. Célestine, J. E. Chavez-Negrete, S. Choi, E. Christiaen, P. Clavijo, L. Colon-Perez, S. Cramer, T. Daniele, E. Dempsey, Y. Diao, A. Doelemeyer, D. Dopfel, L. Dvořáková, C. Falfán-Melgoza, F. F. Fernandes, C. F. Fowler, A. Fuentes-Ibañez, C. Garin, E. Gelderman, C. E. Golden, C. C. Guo, M. J. Henckens, L. A. Hennessy, P. Herman, N. Hofwijks, C. Horien, T. M. Ionescu, J. Jones, J. Kaesser, E. Kim, H. Lambers, A. Lazari, S.-H. Lee, A. Lillywhite, Y. Liu, Y. Y. Liu, A. López-Castro, X. López-Gil, Z. Ma, E. MacNicol, D. Madularu, F. Mandino, S. Marciano, M. J. McAuslan, P. McCunn, A. McIntosh, X. Meng, L. Meyer-Baese, S. Missault, F. Moro, D. Naessens, L. J. Nava-Gomez, H. Nonaka, J. J. Ortiz, J. Paasonen, L. M. Peeters, M. Pereira, P. D. Perez, M. Pompilus, M. Prior, R. Rakhmatullin, H. M. Reimann, J. Reinwald, R. T. de Rio, A. Rivera-Olvera, D. Ruiz-Pérez, G. Russo, T. J. Rutten, R. Ryoke, M. Sack, P.

Salvan, B. G. Sanganahalli, A. Schroeter, B. J. Seewoo, E. Selingue, A. Seuwen, B. Shi, N. Sirmpilatze, J. A. Smith, C. Smith, F. Sobczak, P. J. Stenroos, M. Straathof, S. Strobelt, A. Sumiyoshi, K. Takahashi, M. E. Torres-García, R. Tudela, M. van den Berg, K. van der Marel, A. T. van Hout, R. Vertullo, B. Vidal, R. M. Vrooman, V. X. Wang, I. Wank, D. J. Watson, T. Yin, Y. Zhang, S. Zurbruegg, S. Achard, S. Alcauter, D. P. Auer, E. L. Barbier, J. Baudewig, C. F. Beckmann, N. Beckmann, G. J. Becq, E. L. Blezer, R. Bolbos, S. Boretius, S. Bouvard, E. Budinger, J. D. Buxbaum, D. Cash, V. Chapman, K.-H. Chuang, L. Ciobanu, B. Coolen, J. W. Dalley, M. Dhenain, R. M. Dijkhuizen, O. Esteban, C. Faber, M. Febo, K. W. Feindel, G. Forloni, J. Fouquet, E. A. Garza-Villarreal, N. Gass, J. C. Glennon, A. Gozzi, O. Gröhn, A. Harkin, A. Heerschap, X. Helluy, K. Herfert, A. Heuser, J. R. Homberg, D. J. Houwing, F. Hyder, G. D. Ielacqua, I. O. Jelescu, H. Johansen-Berg, G. Kaneko, R. Kawashima, S. D. Keilholz, G. A. Keliris, C. Kelly, C. Kerskens, J. Y. Khokhar, P. C. Kind, J.-B. Langlois, J. P. Lerch, M. A. López-Hidalgo, D. Manahan-Vaughan, F. Marchand, R. B. Mars, G. Marsella, E. Micotti, E. Muñoz-Moreno, J. Near, T. Niendorf, W. M. Otte, P. Pais, W.-J. Pan, R. A. Prado-Alcalá, G. L. Quirarte, J. Rodger, T. Rosenow, C. S. Baptista, A. Sartorius, S. J. Sawiak, T. W. Scheenen, N. Shemesh, Y.-Y. I. Shih, A. Shmuel, G. Soria, R. Stoop, G. J. Thompson, S. M. Till, N. Todd, A. Van Der Linden, A. van der Toorn, G. A. van Tilborg, C. Vanhove, A. Veltien, M. Verhoye, L. Wachsmuth, W. Weber-Fahr, P. Wenk, X. Yu, V. Zerbi, N. Zhang, B. B. Zhang, L. Zimmer, G. A. Devenyi, M. M. Chakravarty, A. Hess, A consensus protocol for functional connectivity analysis in the rat brain. *Nat. Neurosci.* **26**, 673–681 (2022).

72. C. Grimm, N. Wenderoth, V. Zerbi, An optimized protocol for assessing changes in mouse whole-brain activity using opto-fMRI. *STAR Protoc.* **3**, 101761 (2022).

73. T. D. Ambrisko, Y. Hikasa, Neurohormonal and metabolic effects of medetomidine compared with xylazine in beagle dogs. *Can. J. Vet. Res.* **66**, 42–49 (2002).

74. C. Suzuki, M. Kosugi, Y. Magata, Conscious rat PET imaging with soft immobilization for quantitation of brain functions: Comprehensive assessment of anesthesia effects on cerebral blood flow and metabolism. *EJNMMI Res.* **11**, 46 (2021).

75. L. Laaksonen, M. Kallioinen, J. Långsjö, T. Laitio, A. Scheinin, J. Scheinin, K. Kaisti, A. Maksimow, R. E. Kallionpää, V. Rajala, J. Johansson, O. Kantonen, M. Nyman, S. Sirén, K. Valli, A. Revonsuo, O. Solin, T. Vahlberg, M. Alkire, H. Scheinin, Comparative effects of dexmedetomidine, propofol, sevoflurane, and S-ketamine on regional cerebral glucose metabolism in humans: A positron emission tomography study. *Br. J. Anaesth.* **121**, 281–290 (2018).

76. K. Masamoto, M. Fukuda, A. Vazquez, S. G. Kim, Dose-dependent effect of isoflurane on neurovascular coupling in rat cerebral cortex. *Eur. J. Neurosci.* **30**, 242–250 (2009).

77. N. K. Logothetis, J. Pauls, M. Augath, T. Trinath, A. Oeltermann, Neurophysiological investigation of the basis of the fMRI signal. *Nature* **412**, 150–157 (2001).

78. F. Zhao, T. Jin, P. Wang, S. G. Kim, Isoflurane anesthesia effect in functional imaging studies. *Neuroimage* **38**, 3–4 (2007).

79. T. Maekawa, C. Tommasino, H. M. Shapiro, J. Keifer-Goodman, R. W. Kohlenberger, Local cerebral blood flow and glucose utilization during isoflurane anesthesia in the rat. *Anesthesiology* **65**, 144–151 (1986).

80. M. Ueki, G. Mies, K. A. Hossmann, Effect of alpha-chloralose, halothane, pentobarbital and nitrous oxide anesthesia on metabolic coupling in somatosensory cortex of rat. *Acta Anaesthesiol. Scand.* **36**, 318–322 (1992).

81. N. J. Maandag, D. Coman, B. G. Sanganahalli, P. Herman, A. J. Smith, H. Blumenfeld, R. G. Shulman, F. Hyder, Energetics of neuronal signaling and fMRI activity. *Proc. Natl. Acad. Sci. U.S.A.* **104**, 20546–20551 (2007).

82. C. Alonso Bde, T. Makarova, A. Hess, On the use of α-chloralose for repeated BOLD fMRI measurements in rats. *J. Neurosci. Methods* **195**, 236–240 (2011).

83. K. M. Garrett, J. Gan, Enhancement of γ-aminobutyric $acid_A$ receptor activity by α-chloralose. *J. Pharmacol. Exp. Ther.* **285**, 680–686 (1998).

84. E. Kumamoto, Y. Murata, Enhancement by lanthanide of general anesthetic-induced GABAA-receptor current in rat septal cholinergic neurons in culture. *J. Neurophysiol.* **75**, 2294–2299 (1996).

85. M. Massott, V. G. Longo, α-Chloralose and the central dopaminergic system. *J. Pharm. Pharmacol.* **30**, 667 (1978).

86. N. Montemurro, N. Aliaga, P. Graff, A. Escribano, J. Lizana, New targets and new technologies in the treatment of Parkinson's disease: A narrative review. *Int. J. Environ. Res. Public Health* **19**, 8799 (2022).

87. C. E. Bass, V. P. Grinevich, Z. B. Vance, R. P. Sullivan, K. D. Bonin, E. A. Budygin, Optogenetic control of striatal dopamine release in rats. *J. Neurochem.* **114**, 1344–1352 (2010).

88. G. Paxinos and C. Watson, *The Rat Brain in Stereotaxic Coordinates*. (Academic Press, San Diego; 1998).

89. K. Hamacher, H. H. Coenen, G. Stöcklin, Efficient stereospecific synthesis of no-carrier-added 2-[^{18}F]-fluoro-2-deoxy-D-glucose using aminopolyether supported nucleophilic substitution. *J. Nucl. Med.* **27**, 235–238 (1986).

90. J. A. Disselhorst, D. F. Newport, A. M. Schmid, F. P. Schmidt, C. Parl, C. C. Liu, B. J. Pichler, J. G. Mannheim, NEMA NU 4-2008 performance evaluation and MR compatibility tests of an APD-based small animal PET-insert for simultaneous PET/MR imaging. *Phys. Med. Biol.* **67**, 045015 (2022).

91. W. K. Schiffer, M. M. Mirrione, A. Biegon, D. L. Alexoff, V. Patel, S. L. Dewey, Serial microPET measures of the metabolic reaction to a microdialysis probe implant. *J. Neurosci. Methods* **155**, 272–284 (2006).

92. R. Henson, "Chapter 15 - Efficient experimental design for fMRI" in *Statistical Parametric Mapping*, K. Friston, J. Ashburner, S. Kiebel, T. Nichols, W. Penny, Eds. (Academic Press, 2007), pp. 193–210.

93. M. Brett, J.-L. Anton, R. Valabregue, J.-B. Poline, “Region of interest analysis using an SPM toolbox” in *8th Internation Conference on Functional Mapping of the Human Brain*. (CD-ROM in NeuroImage, 2002), pp. 497.

94. K. J. Friston, E. Zarahn, O. Josephs, R. N. Henson, A. M. Dale, Stochastic designs in event-related fMRI. *Neuroimage* **10**, 607–619 (1999).

95. A. Eklund, T. E. Nichols, H. Knutsson, Cluster failure: Why fMRI inferences for spatial extent have inflated false-positive rates. *Proc. Natl. Acad. Sci. U.S.A.* **113**, 7900–7905 (2016).

96. F. J. López-González, J. Silva-Rodríguez, J. Paredes-Pacheco, A. Niñerola-Baizán, N. Efthimiou, C. Martín-Martín, A. Moscoso, Á. Ruibal, N. Roé-Vellvé, P. Aguiar, Intensity normalization methods in brain FDG-PET quantification. *Neuroimage* **222**, 117229 (2020).

97. L. Rischka, G. M. Godbersen, V. Pichler, P. Michenthaler, S. Klug, M. Klöbl, V. Ritter, W. Wadsak, M. Hacker, S. Kasper, R. Lanzenberger, A. Hahn, Reliability of task-specific neuronal activation assessed with functional PET, ASL and BOLD imaging. *J. Cereb. Blood Flow Metab.* **41**, 2986–2999 (2021).

98. M. J. McKeown, L. K. Hansen, T. J. Sejnowsk, Independent component analysis of functional MRI: What is signal and what is noise? *Curr. Opin. Neurobiol.* **13**, 620–629 (2003).

99. A. Hyvärinen, E. Oja, Independent component analysis: Algorithms and applications. *Neural Netw.* **13**, 411–430 (2000).

100. V. D. Calhoun, V. K. Potluru, R. Phlypo, R. F. Silva, B. A. Pearlmutter, A. Caprihan, S. M. Plis, T. Adalı, Independent component analysis for brain FMRI does indeed select for maximal independence. *PLOS ONE* **8**, e73309 (2013).

101. A. Hyvärinen, E. Oja, A fast fixed-point algorithm for independent component analysis. *Neural Comput.* **9**, 1483–1492 (1997).

102. V. D. Calhoun, T. Adali, G. D. Pearlson, J. J. Pekar, A method for making group inferences from functional MRI data using independent component analysis. *Hum. Brain Mapp.* **14**, 140–151 (2001).

103. A. Hyvärinen, Fast and robust fixed-point algorithms for independent component analysis. *IEEE Trans. Neural Netw.* **10**, 626–634 (1999).

104. A. Nieto-Castanon, *Handbook of fcMRI Methods in CONN* (Hilbert Press, 2020).

105. S. A. Rombouts, F. Barkhof, F. G. Hoogenraad, M. Sprenger, P. Scheltens, Within-subject reproducibility of visual activation patterns with functional magnetic resonance imaging using multislice echo planar imaging. *Magn. Reson. Imaging* **16**, 105–113 (1998).

106. W. C. Machielsen, S. A. Rombouts, F. Barkhof, P. Scheltens, M. P. Witter, FMRI of visual encoding: Reproducibility of activation. *Hum. Brain Mapp.* **9**, 156–164 (2000).

107. L. R. Dice, Measures of the amount of ecologic association between species. *Ecology* **26**, 297–302 (1945).

108. M. E. Weaverdyck, M. D. Lieberman, C. Parkinson, Tools of the trade multivoxel pattern analysis in fMRI: A practical introduction for social and affective neuroscientists. *Soc. Cogn. Affect. Neurosci.* **15**, 487–509 (2020).

109. T. J. Adkins, T. G. Lee, Reward modulates cortical representations of action. *Neuroimage* **228**, 117708 (2021).

110. L. Grosenick, B. Klingenberg, K. Katovich, B. Knutson, J. E. Taylor, Interpretable whole-brain prediction analysis with GraphNet. *Neuroimage* **72**, 304–321 (2013).

111. V. Michel, A. Gramfort, E. Eger, G. Varoquaux, B. Thirion, “A comparative study of algorithms for intra- and inter-subjects fMRI decoding” in *Machine Learning and Interpretation in Neuroimaging*, G. Langs, I. Rish, M. Grosse-Wentrup, B. Murphy, G. Langs, I. Rish, M. Grosse-Wentrup, B. Murphy, Eds. (Springer, 2012), pp. 1–8.

112. C. Allefeld, K. Görgen, J.-D. Haynes, Valid population inference for information-based imaging: From the second-level *t*-test to prevalence inference. *Neuroimage* **141**, 378–392 (2016).

113. R. Gilron, J. Rosenblatt, O. Koyejo, R. A. Poldrack, R. Mukamel, What's in a pattern? Examining the type of signal multivariate analysis uncovers at the group level. *Neuroimage* **146**, 113–120 (2017).

114. M. T. Todd, L. E. Nystrom, J. D. Cohen, Confounds in multivariate pattern analysis: Theory and rule representation case study. *Neuroimage* **77**, 157–165 (2013).

115. S. M. Helfinstein, T. Schonberg, E. Congdon, K. H. Karlsgodt, J. A. Mumford, F. W. Sabb, T. D. Cannon, E. D. London, R. M. Bilder, R. A. Poldrack, Predicting risky choices from brain activity patterns. *Proc. Natl. Acad. Sci. U.S.A.* **111**, 2470–2475 (2014).

116. K. Izuma, K. Shibata, K. Matsumoto, R. Adolphs, Neural predictors of evaluative attitudes toward celebrities. *Soc. Cogn. Affect. Neurosci.* **12**, 382–390 (2016).

117. J. Jiang, C. Summerfield, T. Egner, Visual prediction error spreads across object features in human visual cortex. *J. Neurosci.* **36**, 12746–12763 (2016).

118. J. Kim, J. Wang, D. H. Wedell, S. V. Shinkareva, Identifying core affect in individuals from fMRI responses to dynamic naturalistic audiovisual stimuli. *PLOS ONE* **11**, e0161589 (2016).

119. Q. Wang, B. Cagna, T. Chaminade, S. Takerkart, Inter-subject pattern analysis: A straightforward and powerful scheme for group-level MVPA. *Neuroimage* **204**, 116205 (2020).

120. E. Olivetti, S. Veeramachaneni, E. Nowakowska, Bayesian hypothesis testing for pattern discrimination in brain decoding. *Pattern Recognit.* **45**, 2075–2084 (2012).

121. K. H. Brodersen, J. Daunizeau, C. Mathys, J. R. Chumbley, J. M. Buhmann, K. E. Stephan, Variational Bayesian mixed-effects inference for classification studies. *Neuroimage* **76**, 345–361 (2013).

122. J. Stelzer, Y. Chen, R. Turner, Statistical inference and multiple testing correction in classification-based multi-voxel pattern analysis (MVPA): Random permutations and cluster size control. *Neuroimage* **65**, 69–82 (2013).

123. S. Whitfield-Gabrieli, A. Nieto-Castanon, Conn: A functional connectivity toolbox for correlated and anticorrelated brain networks. *Brain Connect.* **2**, 125–141 (2012).
